# Tailoring renewable materials via plant biotechnology

**DOI:** 10.1186/s13068-021-02010-z

**Published:** 2021-08-05

**Authors:** Lisanne de Vries, Sydne Guevara-Rozo, MiJung Cho, Li-Yang Liu, Scott Renneckar, Shawn D. Mansfield

**Affiliations:** 1 Department of Wood Science, Faculty of Forestry, University of British Columbia, Vancouver, BC V6T 1Z4 Canada; 2 US Department of Energy (DOE) Great Lakes Bioenergy Research Center, the Wisconsin Energy Institute, University of Wisconsin - Madison, Madison, WI 53726 USA

**Keywords:** Plant biotechnology, Plant cell walls, Biomaterials, Biorefinery

## Abstract

Plants inherently display a rich diversity in cell wall chemistry, as they synthesize an array of polysaccharides along with lignin, a polyphenolic that can vary dramatically in subunit composition and interunit linkage complexity. These same cell wall chemical constituents play essential roles in our society, having been isolated by a variety of evolving industrial processes and employed in the production of an array of commodity products to which humans are reliant. However, these polymers are inherently synthesized and intricately packaged into complex structures that facilitate plant survival and adaptation to local biogeoclimatic regions and stresses, not for ease of deconstruction and commercial product development. Herein, we describe evolving techniques and strategies for altering the metabolic pathways related to plant cell wall biosynthesis, and highlight the resulting impact on chemistry, architecture, and polymer interactions. Furthermore, this review illustrates how these unique targeted cell wall modifications could significantly extend the number, diversity, and value of products generated in existing and emerging biorefineries. These modifications can further target the ability for processing of engineered wood into advanced high performance materials. In doing so, we attempt to illuminate the complex connection on how polymer chemistry and structure can be tailored to advance renewable material applications, using all the chemical constituents of plant-derived biopolymers, including pectins, hemicelluloses, cellulose, and lignins.

## Background

Terrestrial colonization of plants from aquatic environments is due, in part, to the development of extracellular cell walls, which conferred rigidity for structural support and facilitated directionality for anisotropic cell growth, as well as providing plants with protection against pests, pathogens, UV light, and diseases [1–5]. Moreover, faced with millions of years of biotic and abiotic stimuli, plants and their associated cell walls have evolved and increased their complexity. Biogeoclimatic pressures, and spatial and temporal regulation of this balancing act further allowed plants to tune features, such as cell shape and cell growth, develop a thick-walled vascular system built of xylem vessels and fibres, and thereby facilitated appropriate form and function to combat a milieu of physiological challenges [6, 7]. Cell walls are more than just supportive and protective features, however, as they are also critically involved in processes, such as cell-to-cell adhesion, water transport, and communication [8]. Consequently, the composition of the cell wall matrix varies between tissues and developmental stages. These specialized features largely manifest from the complex structure and composition of the individual wall polymers, the covalent and non-covalent interactions among them, and their architectural arrangement in individual plant species, facilitating specialized structure–function relationships [9]. In nature, this allows plants actively grow to more than a 100 m in height, as demonstrated by the redwood, Hyperion. The same lightweight, foam-like architecture has been exploited for millenia by humans in building structures of impressive height from these natural materials that have exceptional strength to weight and stiffness to weight ratios.

Cell walls are divided into primary cell walls (PCWs) and secondary cell walls (SCWs), based on differences in their composition and the timing of their developmental deposition. The major chemical constituents of PCWs include cellulose (~ 25%) along with hemicelluloses (~ 30%), specifically xyloglucans, and glycosylated proteins (~ 2–5%), and pectins (40%) [10]. The PCW is extremely plastic in nature; this aspect is due to remodelling events that target the cross-linking polymers by proteins, such as expansins, as it is the PCW alone that surrounds the cell during growth and expansion [10]. SCWs are deposited inside PCWs after growth ceases and consist of some common chemical constituents (to primary walls), but also display unique differences [4, 11]. Most notably, SCWs contain lignin (~ 15–30%), a phenolic polymer whose monolignol monomers are derived from phenylalanine and to a limited extent tyrosine [12, 13], different forms of hemicellulose (~ 30–45%; xylan instead of xyloglucan), cellulose (~ 40–60%), and almost no pectinaceous materials. Notably, primary cell walls can also be lignified once they reached their final size and shape, as the compound middle lamella is formed. In addition, there are differences among species, and cell types within these tissues, clearly illustrating the complexity of these unique plant features.

Annually, it has been estimated that in excess of 100 billion metric tons of carbon dioxide are fixed via photosynthesis into plant cell walls [14]. In perspective, there is a 580 Gt budget to mitigate climate change to under 1.5 °C this century  [15]. Clearly, the vast array of plant species and, therefore, cell wall biomass that dominants the terrestrial biomes offers a valuable renewable resource to combat climate change and could be used for the production of goods and materials vital to society, such as fuels, oils, textiles, chemicals, building products, paper and tissue, food, fibre, nanocomposites, gelling and stabilizing agents, and nutra- and pharmaceuticals [16]. The plethora of inherent plant cell wall polymers, therefore, offers unique opportunities to develop effective and innovative uses of plant biomass in a bio-based economy that continues to strive for a transition from a global dependence on fossil fuels, and fossil-derived chemicals and polymers. From this perspective, plants can either be processed from the top down perspective, which involves the subtractive removal of some of the components or geometry to transform the material into a useful substrate (*i.e.*, a solid piece of lumber). Or, plant polymers can be isolated as building blocks and reassembled or processed into larger structures. In one case, the natural cellular structure template can be exploited to infer properties to the final material, and in the latter, nanoscale and molecular components can be isolated and built into structures that avoid natural imperfections (such as a knot).

**Fig. 1:**
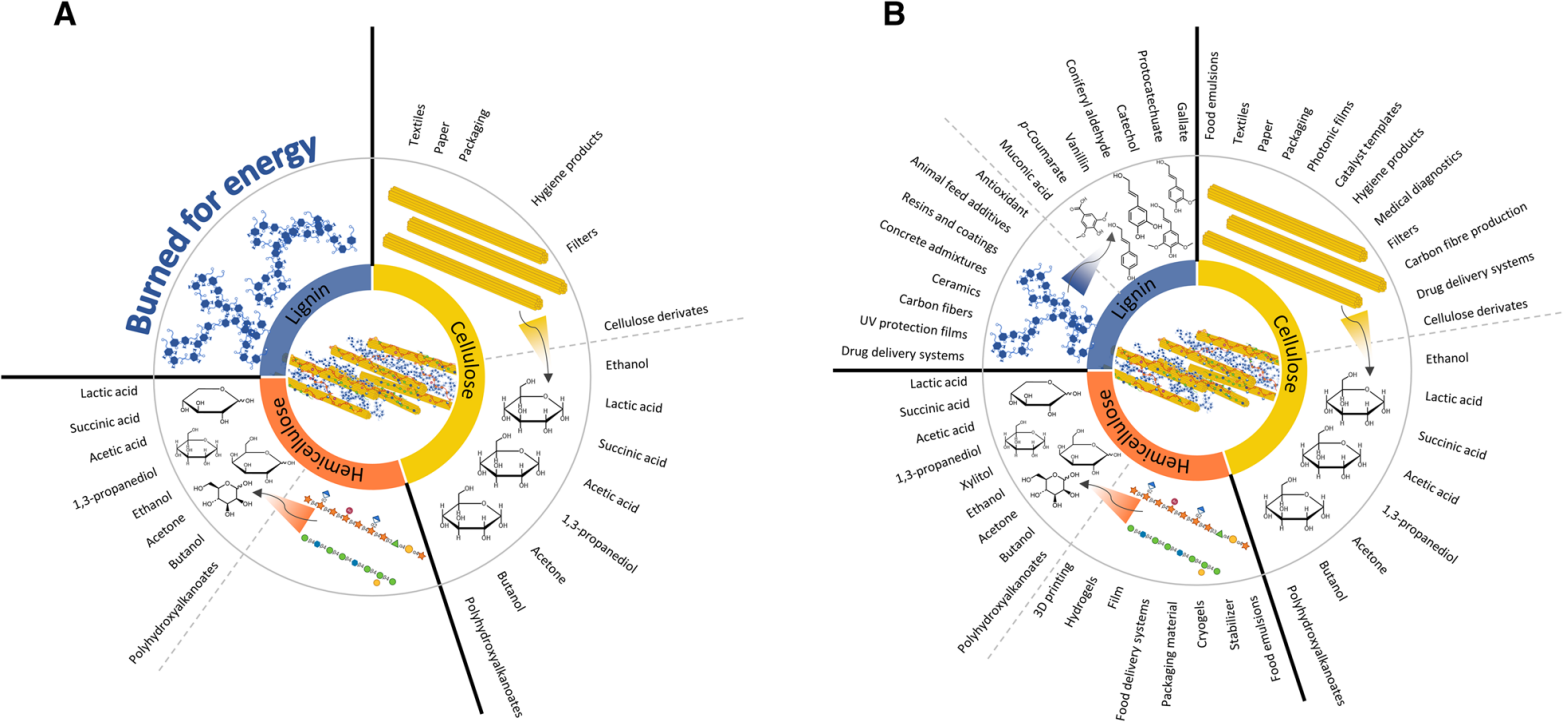
Schematic depiction of the current versus future potential product portfolio of renewables from lignocellulosic biomass. **A** Current situation, where the polymeric cellulose is used for the production of paper and packaging materials, textiles, and cellulose derivatives. The same polysaccharides could be hydrolysed to monosaccharides, which could then be fermented to a plethora of products. Noting that the conversion of polysaccharides to monosaccharides requires harsh chemicals and significant energy, and the required energy could be, completely or partially, derived from burning the phenolic polymer. **B** Strategically, by selection, breeding, or genetically modification of biomass crops, the major plant polymeric fractions would be separated with greater ease, preserving their inherent chemistry and structure, and concurrently increasing the yields of monomers and fermentable products. More importantly, the rescued polymers could be used for the production of high-value products, of which only a few are highlighted in the figure. By combining biotechnology with upstream optimization, innovative downstream separation and material development, lignocellulosic biomass could provide a plethora of green, alternatives for fossil fuels, solidifying the bio-based economy of the future. Pectin is not noted in this figure, as their fractional weight in lignocellulosic biomass is rather small (yet not of any less importance)

Society’s overreliance on petroleum has significantly impacted the globe, and alternatives are currently being sought with the recognition for the need for climate justice solutions. With the potential to offer a reduced carbon footprint, there has been a paradigm shift towards plant and fibre resources for fuels, chemicals, polymeric materials, and engineered composites [16]. However, relative to the petroleum industry, where conversion efficiency of crude oil into products can approach 94% [17], in ~ 100 M barrel/day global industry based on International Energy Agency data, biomass conversion requires significant product diversification to complement and offset the array of products derived from petroleum. As such, to achieve enhanced utilization of plant biomass to meet societal needs we need to improve access to the cellulosic components, and importantly maximize the value of the co-products while concomitantly minimizing waste streams (Fig. 1). To achieve such objectives, a combination of upstream optimization, innovative downstream separation and material application development are required, and this, along with plant biomass engineering may collectively offer a means to overcome hurdles that currently exist in preventing adoption of biorefineries.

Lignin and hemicellulose both inherently contribute to the recalcitrance of lignocellulosic biomass [18, 19], and limit access to the cellulosic component dominating biomass, which is directly useful for fibre, polymer, nanoparticles, and biofuel production. By augmenting the amount or altering the composition of lignin and/or hemicellulose, cell wall (biomass) recalcitrance can be impacted. Consequently, efforts to breed, select natural variants, or genetically engineer plant cell wall biosynthesis could improve plant biomass processing efficiency, such as pulping, deconstruction, and/or saccharification (*i.e.*, enzymatic hydrolysis of the cell wall polysaccharides to monosaccharides), as well improve forage digestibility [20, 21]. These processes would make current production of fiber and paper products have a lower environmental footprint as well as open opportunities for nanocellulose isolation and production of functional wood materials. Furthermore, designer plants with specific characteristics, such as altered cell wall architecture (degree of polymerization and/or crystallinity), engineered lignin structures and linkages, and readily hydrolyzable value-added chemical compounds could afford biomass that would have enhanced value to society.

In the late ‘90s and the beginning of ‘00s the Transfer-DNA (T-DNA)-induced mutation library of the model plant Arabidopsis was created, and dramatically increased our fundamental knowledge of secondary cell wall growth and development [22, 23]. Around the same time, RNAinterference (RNAi)-mediated suppression techniques were introduced (which is more efficient and precise compared to the sense and antisense strategy), and offered an easier and more effective biotechnological means to translate the knowledge obtained from Arabidopsis mutant studies to biomass crops [24]. More recently, the advent of CRISPR/Cas9 genome editing technologies has facilitated a means to make knock-out mutations in crops, similar to the array of mutant studies in model plants (*i.e.*, Arabidopsis), and equally importantly in biomass crops [25, 26]. There are several advantages of CRISPR/Cas9 compared to gene silencing, including: (i) it is possible to make knock-out mutants (with the gene silencing strategies in almost every case there is residual expression); (ii) the CRISPR/Cas9 mutants are stable, while downregulation of (a) certain gene(s) could result in phenotypes that are different from plant to plant, from generation to generation, and even between cells [e.g., the ‘patchy’ patterns that are observed in poplar trees with downregulated *Cinnamoyl-CoA Reductase* (*CCR*)] [27]. If it is desired to have residual expression (e.g., to avoid a yield penalty), it would be possible to make a stable knock-down mutant with the specificity of CRISPR/Cas9, as is recently demonstrated for *ccr* in poplar [28]. In addition, finally, (iii) by crossing a CRISPR/Cas9 transformed crop with a wild-type crop it is possible to remove the T-DNA insert, while the mutation(s) remain in the plant’s DNA, and as a consequence should fulfill the non-GMO label. Although, the European Council decided that genome edited plants are still GMOs, which makes it more difficult to get varieties to market in some geographies [29]. However, this legislative framework may soon change [30].

**Fig. 2:**
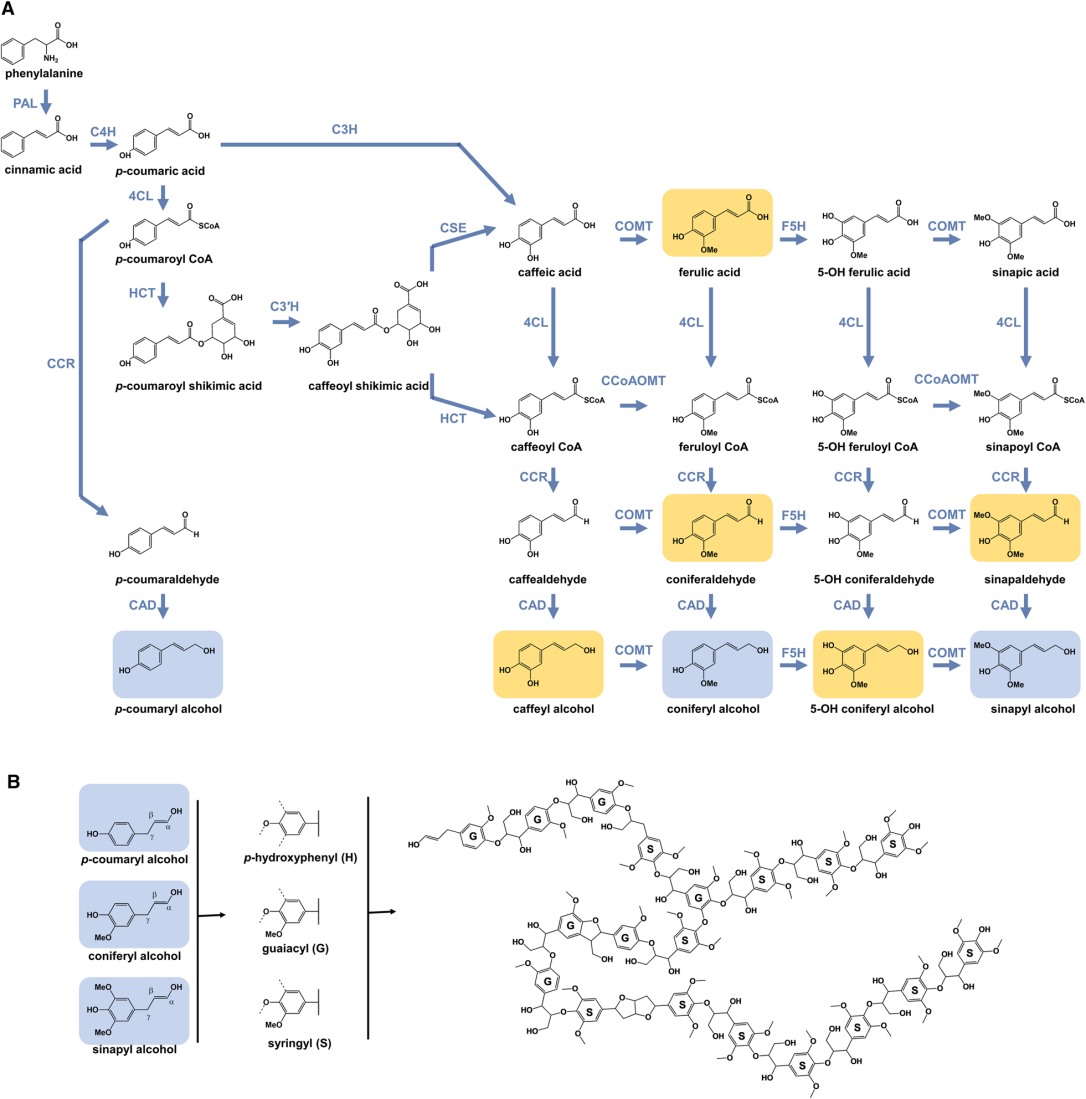
Lignin biosynthesis. **A** In the plant cell (cytosol) the amino acid phenylalanine is converted to the three canonical monolignols (*p*-coumaroyl alcohol, coniferyl alcohol and sinapyl alcohol, indicated in blue) in the “grid-like” phenylpropanoid pathway, where phenylalanine goes through a series of ring (horizontal reactions on the biosynthetic grid) and sidechain (vertical reactions on the biosynthetic grid) modification to produce the monolignols for export to the plant secondary wall for lignification. Some non-canonical monolignols can also be synthesized via the phenylpropanoid pathway, or are intermediates in this pathway (indicated in orange). **B** Following biosynthesis, the monolignols are transported outside the cell, into the apoplastic space, where they are radicalized via laccase and peroxidase enzymes, and these radicals are then incorporated in the lignin polymer via radical coupling. The polymer shown here depicts a poplar lignin, mainly composed of β-aryl-ether (β-*O*-4) bonds (red) and syringyl monomers. 4CL: 4-coumarate:CoA ligase; C3H: *p*-coumarate 3-hydroxylase; C4H: cinnamate 4-hydroxylase; CAD: cinnamyl alcohol dehydrogenase; CCoAOMT: caffeoyl-CoA *O*-methyltransferase; CCR: cinnamoyl-CoA reductase; COMT: caffeic acid *O*-methyltransferase; CSE: caffeoyl shikimate esterase; F5H: ferulate 5-hydroxylase; HCT: hydroxycinnamoyl-CoA shikimate/quinate hydroxycinnamoyl transferase; PAL: phenylalanine ammonia-lysase

Following the successful integration of foreign DNA into plants for pest and pathogen resistance, the early genetically modified plants, particularly trees, were designed to alter lignin content in attempts to improve pulping efficiency in trees, and improve the digestibility and calorific value of forage crops for livestock. To augment lignification, the phenylpropanoid genes were the primary targets, largely attempting to reduce the overall amount of carbon deposited in this cell wall constituent. The initial attempts included overexpression strategies to upregulate genes, and sense and antisense strategies to downregulate genes. For example, via the sense and antisense techniques, the phenylpropanoid genes *Caffeoyl-CoA O-Methyltransferase*, *Caffeic Acid O-Methyltransferase*, and *Cinnamyl Alcohol Dehydrogenase* (*CCoAOMT*, *COMT*, and *CAD*, respectively; Fig. 2) were the early targets for downregulation in poplar trees, with the motivation of improving pulping efficiency and cellulose yield recovery. Reduced *CCoAOMT* expression resulted in a reduction in lignin content and a concomitant increase in syringyl/guaiacyl (S/G) ratio [31], a reduction in *COMT* expression resulted in a reduced S/G ratio [32], and altered (reduced) *CAD* expression lead to the incorporation of aldehydes [33]. An alternate strategy, the overexpression of *Ferulate 5-Hydroxylase* (*F5H*) led to a substantially increased S/G ratio, which consequently resulted in increased chemical (Kraft) pulping efficiency [34, 35]. These biotechnology efforts nicely complement breeding efforts to select for trees with elevated syringyl content [36–38], which are known to display improved pulping properties [39, 40]. Looking forward, these foundational findings help to guide more specialized engineering and breeding efforts in trees.

However, with the resurgence to develop alternative fuels and mitigate global greenhouse gas emissions, the focus changed to increased saccharification efficiency, *i.e.*, the released monosaccharides could be used as a feedstock in the biorefinery. A comprehensive overview of lignin engineered trees and their effect on biomass processing (if tested) was recently reviewed in Chanoca et al. [20]. Along with the research conducted in trees, significant secondary cell wall research/modification has been done in maize, sorghum, and alfalfa, and also in the perennial biomass crops, such as switchgrass, miscanthus, and sugarcane—with the overarching goal of decreasing the cell wall recalcitrance. An overview of genetically modified grasses displaying increased saccharification efficiency has also been recently reviewed by Halpin 2019 (although mostly focusing on downregulation of phenylpropanoid pathway genes) [41].

Although efforts to alter lignin content and composition have shown great success, they often come with an agronomic penalty, and as such in recent years there has been a shift away from focusing solely on saccharification efficiency, and a new concentration on optimizing the recovery and use of multiple chemical fractions of lignocellulosic biomass as a source for high-value materials and chemicals [21]. Such high-value compounds could be derived from the polysaccharides themselves, but also from the aromatic lignin polymer, and ideally from all cell wall constituents. In addition, emerging research in innovative wood utilization has focused on the creation of materials, such as transparent wood which can serve as transparent windows or window frames that perform better than metal titanium alloys currently used in window frame manufacture. This review will provide an overview of the enormous potential to adopt a cradle to grave strategy. We aim to highlight opportunities to take advantage of all of Nature’s building blocks, as the vast products that can be derived from the inherent plant cell wall polymers are highly dependent on the composition of the cell wall, and its innate architecture. Genetic engineering and selective breeding of renewable plant polymers can be targeted in such a way to optimize the production of both commodity chemicals and fuels, but also high value-added polymers and products.

## Lignocellulosic biomass chemical constituents

### Pectin

Pectins play an important role in plant growth and development; they are highly abundant in PCWs of rapidly growing tissues and are one of the most enriched polymers in the middle lamella that joins adjacent cells [42, 43]. During plant development, some pectinaceous polymers are degraded; a process that is essential for cell expansion [44]. For example, alterations in pectin content and/or structure have shown negative effects in organ initiation, anisotropic growth, and cell–cell adhesion [42, 43].

**Fig. 3:**
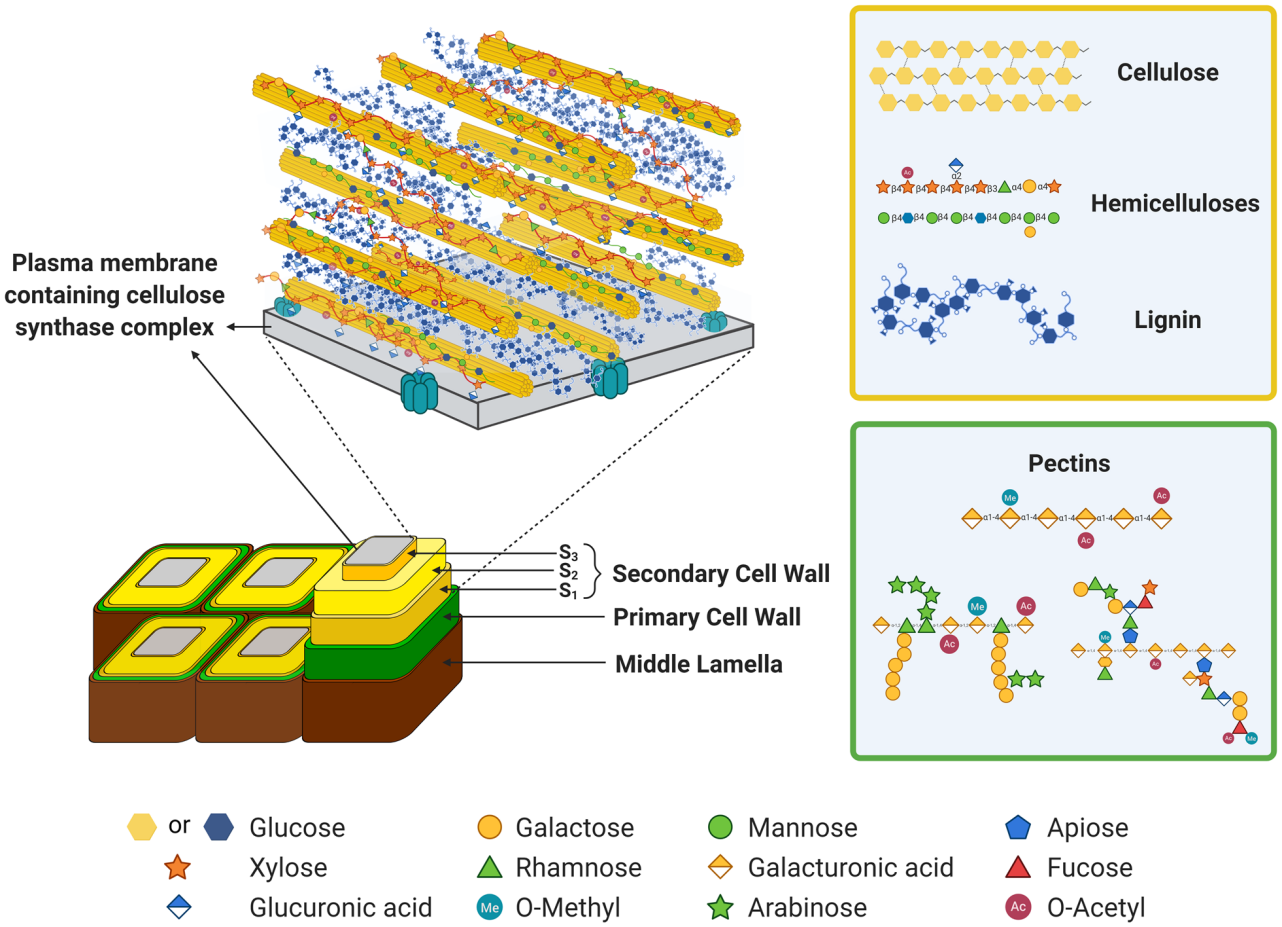
Structural model of the plant cell wall that illustrated a putative model of a plant secondary cell wall. The model shows several cellulose synthase complexes (CSCs) embedded in the plasma membrane depositing cellulose chains into the apoplast as microfibrillar bundles. Hemicelluloses such as glucuronoxylan associate non-covalently to the surfaces of the cellulose microfibrils, while lignification occurs into the cellular voids remaining as the cells enter into programmed cell death. A schematic chemical makeup and linkage of several major and minor cell wall polymers are also shown. Yellow hexagon and blue circle, glucose; dark orange star, xylose; blue–white diamond, glucuronic acid; orange circle, galactose; green triangle, rhamnose; green circle, mannose; orange–white diamond, galacturonic acid; green star, arabinose; blue pentagon, apiose; red triangle, fucose; dark red circle, *O*-acetyl; teal circle, *O*-methyl

Pectin composition (Fig. 3) varies among plants: as a weight fraction of the PCW, pectins account for 35% in dicots, 2–10% in grasses, and 5% in woody tissues [45]. Pectins are classified according to their structures into homogalacturonan (HG), rhamnogalacturonan I (RG-I), and rhamnogalacturonan II (RG-II) [42]. HG is the most abundant pectin in the vast majority plant species, constituting ~ 60% of the pectins in dicot cell walls. It has a simple structure composed of a linear chain of α-1,4-linked galacturonic acid (GalA) residues, which can be C-6 carboxyl methyl-esterified or O-2 and O-3 acetylated. HG can also be decorated with xylose (Xyl) and apiose side chains. RG-I, on the other hand, can account for 7–36% of the PCW in plants depending on origin [46]; its backbone is composed of alternating GalA and rhamnose (Rha) residues that are linked by α-1,2 and α-1,4 bonds, respectively. The RG-I backbone can be decorated with arabinans, galactans, and arabinogalactans side chains which can also contain fucose and ferulic acid moieties [46]. Finally, RG-II is the most complex pectin. It accounts for 1–4% of pectin content in the PCWs of dicots, gymnosperms, and non-gramineous monocots, and for less than 0.1% in grasses [47]. RG-II is composed of 13 different sugars that are linked by multiple glycosidic bonds [42]. At an industrial level, pectins have been widely used in food applications as gelling agents, emulsifiers, and stabilizers [48, 49]. To date, the vast majority of such pectin is derived from fruit peel waste, such as citrus peel and apple pomace.

#### Pectin engineering

Pectins have been identified as important compounds for cell–cell adhesion that can affect the physicomechanical deconstruction of the biomass for the extraction of other cell wall constituents, such as cellulose [50, 51]. Physicomechanical deconstruction (commination) of biomass to a defined particle size contributes significantly to the total feedstock production and supply costs of the biomass conversion [52]. The smaller the particle size, the larger the exposed area and the energy consumption for deconstruction. For example, in the efficient production of corn stover ethanol, particle sizes ranging from 0.5 to 3 mm are required. The physicomechanical reduction from 12 to 3 mm particle sizes can require up to twenty times more energy and generates 25% more ethanol [53, 54]. The high content of pectins in the middle lamella plays an essential role in adjoining adjacent cells and hence, modification to this cell wall fraction offers an excellent target to enhance cell–cell separation, and, therefore, afford reduced energy consumption and concurrently elevate ethanol (bioenergy) generation.

In poplar, the overexpression of *Pectate Lyase1-27* (*PL1-27*), which encodes the protein that is responsible for the cleavage of glycosidic bonds between the GalA units of HG, increased xylose and glucose yields by 21% and 7%, respectively, but reduced stem elongation (Table 1) [55]. Enzymatic treatment of delignified wood particles with xylanase and RG-lyase6 (which cleaves RG-I backbone) manifested in complete cell–cell separation [51]. Likewise, transgenic poplar lines expressing Arabidopsis *RG-Lyase6* showed an increase in xylose (4%) and glucose (25%) yields compared to wild-type plants, without affecting plant growth in some of the lines [51]. Tailoring pectins in woody tissues clearly seems to be a potential way to reduce cell–cell adhesion without affecting plant yield, and consequently reducing energy demand required during deconstruction regimes.

**Table 1:** Targeted genetic engineering of plant cell wall chemical constituents for improved industrial utility

Cell wall compound	Gene	Type of modification	Outcomes	Plant	References
Pectin	*PL1-27*	OE	Increased xylose (21%) and glucose (7%) yields	Poplar	[55]
	*RG-lyase6*	OE	Increased xylose (4%) and glucose (25%) yields	Poplar	[51]
	*PMEI2*	OE	Reduced proportion of egg box structures Increased saccharification (40%) yields	Arabidopsis	[58]
	*GAUT4*	RNAi	Reduced HG and RG-I content, calcium, borate, and ferulate cross-linking Increased lignin migration and hemicellulose dissolution	Switchgrass and Poplar	[60,61]
	*GAUT12*	RNAi	Increased glucose release, plant height, and stem radius	Poplar	[62]
Xyloglucan	*XEG2* (*Aspergillus aculeatus*)	OE	Increased stem height and cellulose content Affected development of G-layers Increased glucose (50%) yields Increased cellulose conversion (60%)	Poplar	[79, 81, 82, 95]
	*XTH4* and *XTH9*	KO (T-DNA insertion)	Reduced XET activity Altered xylem cell expansion and production, and secondary cell deposition Increased carbohydrate production (15%)	Arabidopsis	[84]
	*XET16-34*	OE	Stimulated cell expansion in vessel elements Increased overall xyloglucan content	Poplar	[83]
	*BGAL10*	KO (T-DNA insertion)	Reduced β-galactosidase activity against XyG Altered XyG composition and plant growth	Arabidopsis	[104]
Xylan	*ESK1*	KO (T-DNA insertion and induced point mutation)	Dwarf plants Collapsed xylem vessels	Arabidopsis	[106]
	*DARX1*	KO (CRISPR/Cas9 mutation)	Altered arabinoxylan conformation and cellulose microfibril orientation Reduced mechanical stem strength and plant height	Rice	[117]
	*GUX1* and *GUX2*	KO (T-DNA insertion)	Weak stems Increased glucose (30%) release Increased xylose (700%) release Increased ethanol yields	Arabidopsis	[118, 119]
	*ARAF1* and *ARAF2*	OE	Reduced arabinose (up to 25%) content Increased glucose (up to 34%) release	Rice	[126]
	*AT10* from Rice	OE	Reduced ferulic acid levels Increased saccharification efficiency (40%)	Switchgrass	[127]
Mannan	*CSLA2*,*3*,*7*, and *9*	KO (T-DNA insertion)	CSLA 7 is essential for embryogenesis CSLA 2,3,9 reduced glucomannan content without affecting plant growth	Arabidopsis	[144]
	*MUCI10*	KO (T-DNA insertion)	Altered seed mucilage density and cellulose structure	Arabidopsis	[150]
Cellulose	*CesA1* and *9*	KO (T-DNA insertion and point mutation)	Reduced cellulose crystallinity (up to 34%) Increased fermentable sugar release (up to 151%) Reduced anisotropic growth	Arabidopsis	[168, 171, 172]
	*CesA4*, *CesA7-A/B*, and *CesA8-A/B*	RNAi	Reduced cellulose content and plant growth Collapsed vessels and thinner fibre cell walls Stems exhibited reduced mechanical strength	Poplar	[173]
	*RIC1*	OE	Reduced cellulose crystallinity	Arabidopsis	[174]
	*Cel9A6* and *KOR1*	RNAi	Collapsed xylem vessels Reduced cellulose content and crystallinity Reduced stem mechanical strength	Poplar	[176, 177]
	*Cel9A6*	OE	Increased plant growth and fibre cell length Caused male sterility	Arabidopsis	[176]
	*Cel9A1/KOR1*	OE	Reduced cellulose crystallinity Improved glucose yields	Arabidopsis	[179]
	*GH9B1* and *GH9B3*	OE	Reduced cellulose degree polymerization and crystallinity Increased bioethanol yields	Rice	[180]
	SuSy	OE	Increased cellulose content (up to 6%) and crystallinity Increased wood density	Poplar	[185]
	*SUS3*	OE	Reduced cellulose crystallinity and xylose–arabinose proportions Increased stress-induced callose accumulation	Rice	[186]
Lignin	*4CL*	RNAi	Reduced lignin, changed lignin composition, growth impairments	Poplar	[215]
	*HCT*	RNAi	Reduced lignin, changed lignin composition, growth impairments	Poplar, alfalfa	[216, 218, 219]
	*CCR*	RNAi	Reduced lignin, changed lignin composition (incorporation of ferulic acid, increase in acetal bonds), growth impairments	Poplar, maize	[217, 260]
	*C4H*	RNAi	Reduced lignin, changed lignin composition, growth impairments	Alfalfa	[218, 219]
	*C3′H*	RNAi	Reduced lignin, changed lignin composition, growth impairments	Alfalfa	[218, 219]
	*PAL*	RNAi	Reduced lignin content, but no growth impairments	Poplar	[216]
	*Ref8-1 med5a/med5b*	KO (Triple mutant T-DNA insertion)	Restoration of growth of the *ref8-1* (*c3h*), lignin almost completely composed of H units	Arabidopsis	[226]
	*HCHL*	OE	Reduced lignin degree of polymerization, but no differences in biomass yield or lignin amount. Increased saccharification efficiency	Arabidopsis	[229]
	*CHS*	Natural mutant (*C2-Idf*)	Reduced incorporation of tricin in the lignin, lignin was enriched in β–β and β-5 units	Maize	[232]
	*Sfe*	Transposon Mutant	Reduced feruloylation, better forage digestibility	Maize	[237–241]
	*Gt61*	Natural KO mutant (*xax1*)	Reduced arabinosyl substitutions, increased processing efficiency, dwarfed	Rice	[124]
	*COMT*	KO and RNAi	Incorporation of 5-hydroxyconiferyl alcohol, increase in benzodioxane structures in the lignin, but no lignin reductions. Increased saccharification efficiency	Arabidopsis, Poplar	[243, 244]
	*F5H*	OE	Increased S units (around 90% of all lignin monomers), increased monomer yield after hydrogenolysis	Poplar	[40, 258]
	*FMT*	OE	Incorporation of ester linkages in the lignin backbone, increased saccharification efficiency after alkaline pretreatment, increased pulping efficiency	Poplar	[216, 260]
	*PMT*	OE	Incorporation of *p-*coumarate conjugates in the lignin, higher frequency of terminal units with free phenolic groups	Arabidopsis, Poplar	[263, 264]
	*DCS* & *CURS2*	OE	Incorporation of curcumin in the lignin, increased saccharifcation efficiency	Arabidopsis	[265]
	*Cα-dehydrogenase*	OE	Appearance of chemical labile α-keto-β-ether units in the lignin	Arabidopsis	[266]
	*CAD*	RNAi and KO mutants	Increased concentrations of aldehydeS, increased saccharification efficiency	Pine, Arabidopsis, Medicago, Poplar	[230, 243]
	*QsuB*	OE	Reduced lignin concentrations	Arabidopsis	[282]

*OE* overexpression, *RNAi* RNA interference, *KO* gene knockout, *T-DNA* transfer DNA

The genetic modification of methyl group content in pectins have also shown to significantly affect cell–cell adhesion, cell wall porosity, and elasticity [56]. In the apoplast, Pectin Methylesterases (PMEs) catalyze the demethylation of HG and produce HG with free carboxyl groups that can form calcium bonds conformations with other HG chains (known as egg box conformation) [57]. The Ca^2+^-mediated crosslinks among HG chains favour gelation and interfere with the accessibility of hydrolytic enzymes (in a biochemical biorefinery platform), such as cellulases and hemicellulases, during biomass processing [56, 57]. The activity of PMEs is regulated by different mechanisms and involves the action of Pectin Methylesterase Inhibitors (PMEIs) [56]. In Arabidopsis, the overexpression of *AtPMEI2* reduced the content of egg box structures and increased the saccharification yields by as much as 40% compared to wild-type plants [58], suggesting that the alteration of *PMEIs* expression is also a promising way to reduce cell–cell adhesion.

Similar to the Ca^2+^ mediated cross-linking of HGs, RG-II chains can be joined by borate-diol ester bridges [47]. Experiments with boron depletion have shown that RG-II borate mediated crosslinks play an important role in cell integrity and tissue mechanics [47, 59]. There is evidence that suggests that pectins, in addition to having intra-crosslinks, are also covalently joined to other cell wall polymers [60]. The genetic modification of these inter- and intra-crosslinks have shown positive effects in biomass conversion [61, 62]. The downregulation of *Galacturonosyltransferase 4* (*GAUT4*) in switchgrass and poplar reduced HG and RG-I content, calcium, borate, and ferulate cross-linking, and increased hemicellulose dissolution and lignin migration [60, 61]. Together, these genetic changes enhanced both growth and increased sugar release (14–15%) and ethanol yield compared with control plants [61]. Similarly, the downregulation of *GAUT12* in poplar increased glucose release, plant height, and stem radius [62]. Although the nature of pectin-cell wall polymers interactions still remains to be fully elucidated, it represents a powerful target for genetic engineering.

As a biomaterial, the clear advantage that pectinaceous materials offer is their water solubility arising from the abundant carbonyl groups, consequently facilitating the formation of films, hydrogels, and fibres using aqueous systems [48, 63]. A key parameter in this process is solubility, which is impacted by molecular weight (MW). While enzymes can hydrolyze GalA units for better cellulose recovery, as mentioned above, the lower molecular weight pectin fraction has enhanced solubility and can be extracted at higher yields [64]. Furthermore, the substitution of the GalA units impacts material properties. For example, the galacturonate segments on pectin can readily gel with divalent ions—this ionic bridging process has been used to stabilize electrospun pectin nanofibres in tissue engineering applications [65]. Furthermore, when the degree of methoxylation was modified based on pectin type, samples with a higher degree of methoxylation showed enhanced cell adhesion for fibroblast cell lines [66]. In another study the free GalA groups in pectin, with a low methyl ester content, were able to be effectively crosslinked with calcium ions for biocompatible hydrogel formation [65]. Hence, tailoring MW and degree of substitution *in planta* can have large impact on the performance of the pectins in biomaterial applications. These materials are just beginning to be investigated for use in advanced novel materials, such as transient electronics [67], phase change material capsule shells [68], and flame-retardant aerogels [69, 70]. Furthermore, regiospecific modification of polysaccharides, such as ring opening grafting of lactones on pectin [71], offers a new class of amphiphilic polymers based on the degree of substitution of the methyl ester.

### Hemicelluloses

#### Xyloglucan

Xyloglucan (XyG) is the most abundant hemicellulose in the PCW of dicots, gymnosperms, and non-gramineous monocots accounting for 20–30% (w/w; Fig. 3), in grasses, XyG constitutes only 2–10% (w/w) [72, 73]. XyG consists of a β-1,4-linked glucan backbone partially decorated with Xyl substituents at the O-6 position. The XyG backbone and Xyl residues can be further substituted with galactosyl, fucosyl, galactofucosyl, arabinopyranosyl, or arabinofuranosyl groups [72, 73]. XyG can also carry *O*-acetyl groups on the glucan backbone or on the galactosyl or arabinofuranosyl substituents. Based on their structure, most of the XyGs known to date can be classified into two types: fucogalactoxyloglucan and acetoxyloglucan. Fucogalactoxyloglucan is commonly present in most dicot species; it is xylosylated at three of four backbone glucosyl residues and is usually galactosylated and fucosylated. Acetoxyloglucan is common to grasses and is characterized as having xylosylations in two out of four or more glucosyl residues in the backbone [72, 73]. Acetoxyloglucan is often *O*-acetylated in the glucan backbone residues, while fucogalactoxyloglucan is *O*-acetylated on the side-chains [72].

XyGs play an important role in cell growth and differentiation by interacting with cellulose microfibrils in the PCW. It has been proposed that XyG works as a tether allowing the loosening and tightening of cellulose microfibrils during cell maturation [74]. In contrast, in seeds, XyG accounts for up to 42% of the total weight and serves as a storage polysaccharide that provides energy to the seedlings once they have germinated [72]. Industrially, XyG is currently used as a thickening, stabilizing, and gelling agent [75], and has more recently been considered a polysaccharide with potential in textile applications, the pharmaceutical industries, as well as for drug-delivery technologies [76, 77].

##### Xyloglucan engineering

It has been shown that XyG interferes with biomass conversion by interacting with cellulose microfibrils and other polymers in the cell wall [78, 79]. Loosening of XyG has, therefore, been proposed as a strategy to modify and accelerate saccharification of the other dominant cell wall carbohydrates, such as cellulose [78, 79]. Most of the efforts to limit XyG-cell wall polymers interactions have focused on the enzymes that act on XyG in the apoplast, such as Xyloglucan Endotransglycosylase/hydrolase (XTHs; Table 1) [80], that exhibit XyG:endohydrolase (XEH) and XyG:endotransglycosylase (XET) activity which have been shown to alter the length of XyG chains. In poplar, transgenic trees expressing the fungal *Aspergillus aculeatus Xyloglucanase 2* (*AaXEG2*) were shown to possess reduced XyG content, but displayed increased stem height and cellulose content [81, 82]. Poplar trees overexpressing *AaXEG2* had a 50% increase in glucose yields and 60% cellulose conversion [79]. Similarly, modification of XET proteins in Arabidopsis and poplar have increased saccharification yields and significantly alter secondary growth [83, 84]. In contrast, mutants of *xth4* and *xth9* genes, that show reduced XET activity in Arabidopsis, display altered xylem cell expansion, xylem production, and secondary wall deposition [84], and manifest (only in *xth9* mutants) in 15% gains in sugar production and 50% more growth compared with wild-type plants [84].

The enzymatic action of XTH enzymes also contributes to tension wood formation in angiosperm trees, which is a unique xylem formed on the upper side of leaning stems in response to environmental stress. This specialized tissue is characterized as having a highly crystalline cellulose content, often referred to as cellulose-rich, gelatinous-layer (G-layer) [85–88]. Tension wood tissues are largely unlignified and porous, having high sugar conversion yields and, therefore, being of high industrial interest [89]. Although there is controversy about the presence of XyG in tension wood [90, 91], several studies have reported the activity of XET enzymes during the formation of G-layers [87, 92–94]. In poplar, for instance, the overexpression of the fungal endotransglycosylase *AaXEG2* reduced growth strain, suggesting that XETs affect the development of G-layers and the properties of the associated cellulose [95].

Recent studies have shown the possibility of XyG co-crystallization with cellulose microfibrils and the formation of amorphous zones indicating how closely associated XyG is with cellulose surfaces [96]. Adsorption studies using model films to investigate XyG interactions with cellulose have shown entropy driven processes [97]. Further studies, by Esker and co-workers revealed similar affinity to both amorphous and crystalline surfaces of cellulose [98]. This work elucidating XyG adsorption to cellulose follows on the industrial applications of XyG utilized as a sizing agent. Brumer and co-workers reviewed these historical uses of XyG for textile and paper modification and also introduced the novel idea of using XyG as a vector to add functionality to cellulose surfaces [78]. On the application side, inclusion of XyG into cellulose nanocomposites has demonstrated synergistic performance when blended at certain ratios [99]. XyG has also recently been used in 3D printing of biobased polymer blends [100]. With the interest across the scientific community in nanocellulose materials, it is clear that XyG will have an increased role in material development, where aqueous-based modification of cellulose will help create advanced materials without the need for malignant organic solvent processing [100].

There can still be additional targets for XyG modification. For instance, lignocellulosic biomass can be used for the production of hydrogels [101, 102], but the properties of hydrogels have been shown to be affected by changes in the XyG structure. For example, the enzymatic removal of galactosyl side chains from the XyG backbone in biomass was shown to increased gelation and gel strength [101, 103]. In Arabidopsis, β-Galactosidase 10 (AtBGAL10) was shown to be responsible for the majority of β-galactosidase activity against XyG, and its absence significantly altered XyG composition and plant growth [104]. Therefore, such a β-galactosidase may offer a target to effectively modify XyG side chains and thus alter cell wall ultrastructure in such way that carbohydrate gels could be beneficially affected. Hence, the diversity of hetero-polysaccharide structure contributes to the performance and additional studies are required to exploit these polysaccharides in new biomaterial applications.

#### Xylan

Xylans (Fig. 3) play an essential role in cell wall integrity by interacting with cellulose and lignin [105–108]. Xylan constitutes the most abundant hemicellulose in the SCW of dicots, accounting by approximately 20% (w/w). Xylan is composed of repeating β-1,4 xylose residues as a backbone that can be decorated with glucuronic acid (GlcA), (methyl)glucuronic acid ([Me]GlcA), and/or arabinose residues, and often xylans are acylated to various degrees [109, 110]. Xylans can be grouped into several classes, including glucuronoxylans (GX), glucuronoarabinoxylans (GAX), and arabinoxylans (AX) depending on the type and abundance of their decorations.

Glucuronoxylans constitute one of the major components of the SCW in woody and herbaceous dicots, accounting up to 30% of the total dry mass weight. The GX backbone can be decorated with GlcA and 4-*O*-MeGlcA residues and can be partially acetylated at some Xyl units at the C-2 and/or C-3 positions [110, 111]. In some monocots (Alismatales and Asparagales), [Me]GlcA substitutions of GX are often substituted at the O-2 by arabinopyranose residues [112]. Glucuronoarabinoxylans by contrast, are commonly found in grass species and gymnosperms. In grasses, GAX accounts up to 20–40% (w/w) of PCWs and 40–50% (w/w) of SCWs, and its backbone is highly substituted with arabinofuranose (Araf) and GlcA/[Me]GlcA residues [112]. Araf decorations can be found every 5–12 Xyl units, while GlcA/[Me]GlcA substitutions are often found every 5–6 xylose units. Arabinose residues can be further esterified with ferulic or *p*-coumaric acids [110]. Finally, AXs are abundant in grass endosperm walls, where they account for up to 70% (w/w) of the weight [110]. The primary side group in AX backbone is arabinose, which can be decorated with ferulic acid; the AX backbone can carry acetyl groups at the C-2 and C-3 positions [110]. In GX and GAX from SCWs of dicots and gymnosperms a reducing end is present, and this reducing end can consist of a Xyl, Rha, and/or GlcA residues (β-Xyl-1-3-α-Rha-1-2-α-GalA-1-4-β-Xyl) which is thought to be required for normal synthesis of both polymers [105]. At an industrial level xylan has strong potential for the production of packaging materials, hydrogels, and chemicals, such as furfural, xylitol, lactic acid, and ethanol [113].

##### Xylan engineering

Xylan facilitates cell wall polymers interactions among cellulose and lignin [114]. Acetate and GlcA decorations along the xylan backbone are important to permit docking of xylan with cellulose microfibrils, as well as to maintain microfibrils stability and orientation [106]. As such, xylans play an important role in biomass recalcitrance, largely acting as a barrier restricting accessibility of chemicals and enzymes to cellulose, which dominants plant SCWs. Some (thermo)chemical pretreatments can degrade and/or liberate xylan fragments, while the complete breakdown of xylan requires a set of hydrolytic enzymes that increase the economic and metabolic costs of biomass conversion. In switchgrass, the chemical removal of xylan increased the glucose yield between 54 and 84%, while in poplar 31–57% increases were similarly observed [115]. Hence, genetic modification targeting xylan content and structure has strategic benefits to reduce cell wall recalcitrance.

Attempts to reduce xylan content have focused on genes that either form the xylan synthase complex or are related to the synthesis of the reducing end. The mis-regulation of these genes, however, also negatively effects plant growth and stem strength, making their use challenging for plant genetic engineering efforts [105, 116]. In contrast, modification of xylan structure has met with some positive results, ultimately enhancing biomass conversion without affecting growth.

Molecular dynamic simulations and nuclear magnetic resonance (NMR) studies have shown that xylan folds as a twofold screw when it binds to cellulose [107, 114]. To be compatible with cellulose, xylan decorations need to be in an even pattern. An abnormal pattern of acetyl substitutions in the Arabidopsis *eskimo1* (*esk1*) mutant (a member of a large gene family of DUF231 domain proteins) was shown to prevent the “normal” interaction between xylan and cellulose, and manifests in dwarf plants and collapsed xylem vessels (Table 1). Changes in acetyl substituents also alter GlcA pattern substitutions, as these are added to the xylan backbone in an ESK1-dependent xylan acetylation pattern [106]. Acetyl groups on xylan side chains also affect plant growth and are required to maintain cell wall stability [117]. Deacetylase on Arabinosyl Sidechain of Xylan 1 (DARX1) was identified as an enzyme responsible for the deacetylation of arabinosyl residues in rice [117], and disruption of DARX1 changed arabinoxylan conformation and cellulose microfibrils orientation. These mutant plants had a higher content of threefold xylan and a reduction in mechanical stem strength and plant height [117]. Together, these results suggest that genetic modification of acetyltransferases represents a potential strategy to alter cell wall ultrastructure, although their effects in plant growth require further exploration.

Altering the amount of GlcA decorations has also been explored. In addition to maintaining the two- or threefold conformations, GlcA substitutions appear to contribute to cell wall stability by binding single xylan chains via Ca^2+^-mediated crosslinks [107]. In angiosperms, GlcA decorations are added to 1 in every 8 xylose units, while in gymnosperms, the ratio is 1 in every 6. Modification to these substitutions has been explored by altering the *Glucuronyltransferase* (*GUX1* and *GUX2*) gene expression. In Arabidopsis, mutation of *gux1* and *gux2* causes weak stems without affecting growth or xylem vessels integrity [118]. Moreover, Arabidopsis *gux1 gux2* double mutants showed a 30% increase in glucose release and 700% more xylose release than wild-type plants [119]. Other glycosyltransferases have been identified in commercially relevant plants, such as spruces (*Picea glauca*; PgGUX, Gene Bank: BT111578.1), and shown to be responsible for adding GlcA residues to xylan in vitro [119].

Likewise, it is thought that arabinose substitutions play an important role in stabilizing polymer associations by their ferulate decorations. It has been observed that AXs with a low degree of arabinose substitutions tend to be more folded and entangled than highly substituted AXs. The self-association among xylan chains with a low degree of arabinose substitution decreased xylan solubility and increased plant stem stiffness [120, 121]. Ferulate decorations of arabinosyl substitutions forms cross-links between adjacent AX chains and lignin in grasses [122, 123]. The removal of these residues from xylan side chains has shown to positively affect biomass process ability in rice and switchgrass [124–127]. In rice, for example, the overexpression of two *Arabinofuranosidases* (*OsARAF1* and *OsARAF3*), increased glucose releases up to 34% [126]. Similarly, the heterologous expression of rice *OsAT10* (a putative *p*-coumaroyl coenzyme A transferase that alters the content of ferulic acid and *p*-coumaric acid in GAX) in switchgrass, reduced ferulic acid levels and increased saccharification efficiency by 40% without having negative effects in plant growth [127]. Hence, the modification of xylan decorations is a potential target to increase saccharification, as well as the isolation of other major plant polymers during the fractionation of biomass.

**Fig. 4:**
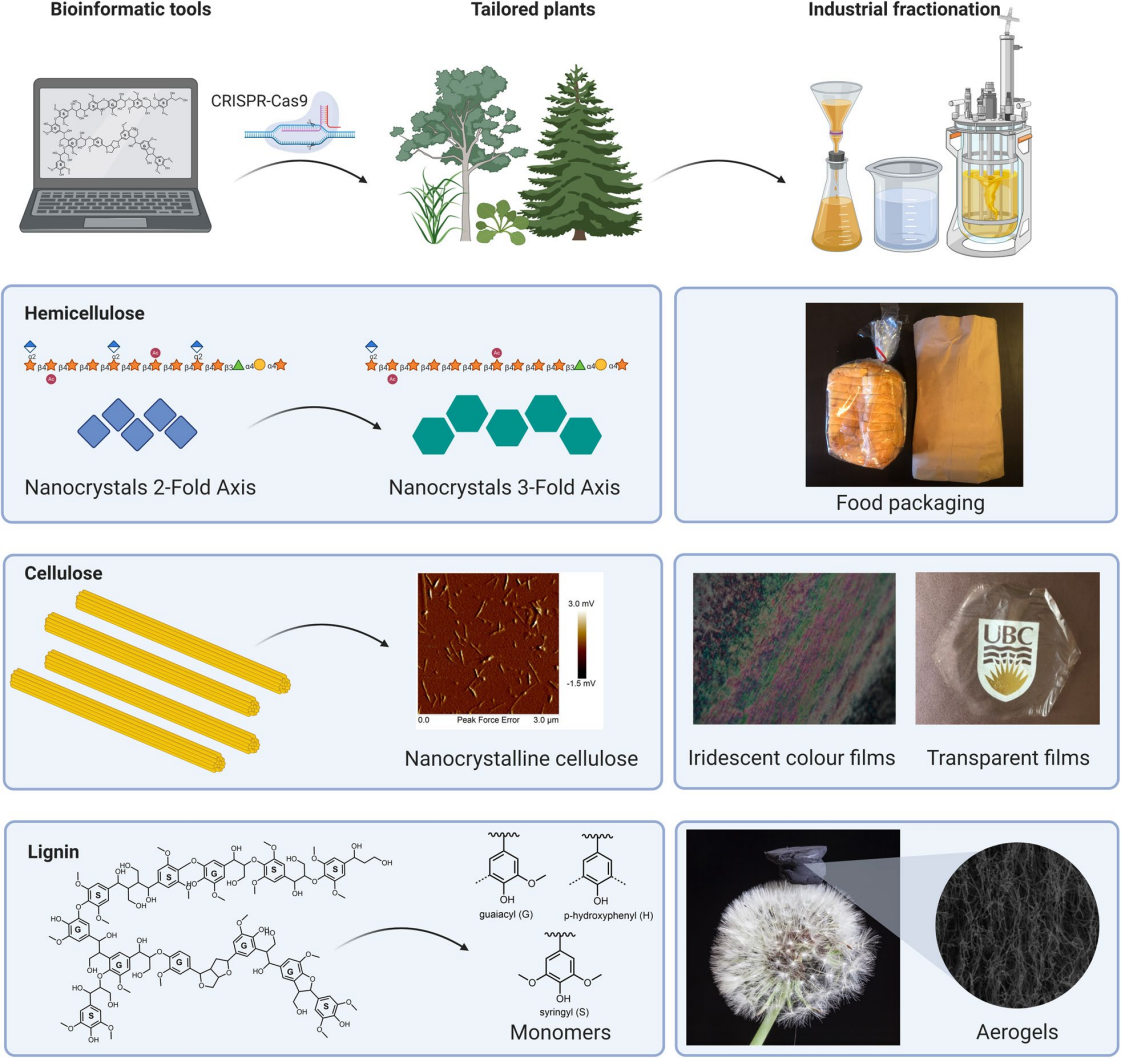
Bioinfomatic tools can inform biotechnologists to create plants with preferred chemistries and ultrastructures for industrial fractionation and utilization. The schematic figure demonstrates potential applications for all three of the major secondary cell wall polymers. Hemicelluloses with less substitution can be readily crystallized and used to generate oxygen barrier films; cellulose crystallinity and degree of polymerization can be optimized for nanocellulose isolation for the generation of photonic films of chiral nematic structures of cellulose nanocrystals or high transparency nanofibrillated cellulose materials; lignin with unique monomers or labile linkages can be synthesized to facilitate the isolation of both monomers and/or lignin polymer fractions tailored for spinning fibre and conversion into aerogels and cryogels

Converting hemicelluloses into new materials provides a unique route to bring additional value to a biorefinery. As fractionation is important for biomass utilization, xylan can be isolated in significant quantity in different pretreatment processes, such as alkali extraction or hot water extraction. However, many chemical pretreatments can hydrolyze the pendant side chains of hemicelluloses, such as arabinans [128] or even deacetylate the backbone [129]. Understanding the functionality that controls material performance of isolated xylan is important to design effective deconstruction strategies to preserve value, maintaining specific structures, and even enhance these structures *in planta*. Outside of breaking xylan into small molecules for xylitol production [130], xylan has been explored for more sustainable packaging materials [131]. This application has shown xylan providing relatively low oxygen permeability values as a film material (Fig. 4). Both arabinoglucuronoxylan [132] and glucuronoxylans [133] from softwoods and hardwoods, respectively, were studied in these packaging applications. Significant differences in mechanical integrity was observed with the former being able to be cast into flexible films and the latter requiring a plasticizer. The spruce arabinoglucuronoxylan had similar behaviour to oatspelt arabinoxylan that required plasticizer for cohesive film formation. Hence, slight differences in structure can have a dramatic impact on the performance—it was claimed that spruce-derived xylans had a proper balance of GlcA and pendent arabinose groups [132]. The films showed limited crystallinity, where chain substituents play a critical role in chain interactions [134] and assembly [135].

The crystallization of xylan has been studied sporadically since the 1940s when Yundt attempted to isolate extremely purified xylan via sequential autoclaving cycles after heating samples in water [136]. Xylan was solubilized and then crystallized as the polymer was cooled from a supersaturated solution. Side groups of xylan that would interrupt packing of the chains of crystals [137] were cleaved by successful purification cycles resulting in the xylan forming a threefold axis crystal hydrate structure [138]. Chanzy and researchers further investigated such structures and used electron diffraction to provide the current state of knowledge on xylan crystals [139]. Purification of xylan leading to crystals suggests the possibility of using biotechnology to remove specific side groups common to xylan that would aid in controlling the formation of xylan single crystals in aqueous suspensions. In addition, xylan has been shown to co-crystalize with DMSO in a twofold axis that creates needles, such as crystals [140]. If optimized, these highly anisotropic crystals would provide an interesting platform for hemicelluloses to be used in nanotechnology applications, such as photonic films.

#### Mannan

Mannans (Fig. 3) account for 3–5% (w/w) of the cell wall composition of hardwoods and 15–25% (w/w) of the biomass in gymnosperms [109, 110]. Mannans are divided into four distinct classes according to their backbone and side-chain substituents: linear mannan, glucomannan, galactomannan, and galactoglucomannan [109]. The backbone of linear mannans consists entirely of mannose, while the backbone of galactomannans consists of mannose with galactosyl substitutions. These polymers are mainly found in seeds, and two of the major sources available industrially are ivory nut and guar seeds. Glucomannans have a backbone of mannose and glucose residues that are often acetylated. They are hydrophilic polysaccharides found in lignified walls of woody tissues, mostly hardwoods [86, 141]. One of the most important natural sources of glucomannans is the konjac plant [142, 143]. Finally, galactoglucomannans have a backbone of mannose and glucose with galactosyl substitutions, and galactoglucomannans can also be acetylated to some degree, depending on species and tissue.

Physiologically, mannans are vital for plants as they act as signalling molecules during embryogenesis and tissue differentiation [144, 145]. In the seed endosperms, they provide protection against dehydration and also provide a simple source of carbon during early seedling development [146, 147]. Mannans are also predicted to be essential for cell wall stability by interacting with other polymers; however, these interactions are poorly understood [148, 149]. Industrially, mannans are used in the production of gums (e.g., guar gum), food stabilizers, and thickening agents [142, 143], and are considered a potential source for the production of packaging materials and hydrogels [142, 143].

##### Mannan engineering

Recent studies have shown that mannans directly bind to cellulose microfibrils [149], and have been suggested to cross-link to lignin via ether bonds [148]. However, the role of these interactions in cell wall stability and biomass conversion have not been widely explored. In Arabidopsis, a mutation in *glucomannan 4-beta-mannosyltransferase 7* (*csla7*) demonstrated that this gene is essential for embryogenesis [144]. In contrast, mutations in *csla2*, *csla3*, and *csla9* reduced glucomannan content without affecting plant growth (Table 1) [144].

It is thought that mannan substitutions may play an important role in altering cellulose structure and seed mucilage density [147, 150]. The degree of galactosyl substitutions has been shown to be important for mannan water solubility, as unsubstituted mannan chains can form crystalline polymers by interacting via hydrogen bonds. Galactose branches limit the self-association of mannan chains and hence increase its solubility [151]. Future studies should explore the role of mannan in cell wall stability by determining how its content and side chain structures affect the interactions among the different cell wall polymers.

As described, Nature has developed mannans for a critical function in the performance of cell walls and seed coats, with various polymer architectures, degrees of acetylation, and substitution. These materials have an integrated assembly with hetero-polysaccharides, yet individually some mannans such as galactomannans have found important use in industrial applications, such as food emulsions [152]. In addition, mannans such as spruce derived galactoglucomannan have been evaluated for food delivery systems [153, 154], demonstrating their applicability in stabilizing omega fatty acids and used for drinkable yogurt. In another study, residual lignin from covalent lignin–carbohydrate linkages with galactoglucomannan was shown to help provide amphiphilicity to the polymer and stabilization to oil-in-water emulsions [155]. With many isolation processes for wood cell wall polysaccharides, such as glucuronoxylan and galactoglucomannan, it can be difficult to isolate a lignin free hetero-polysaccharide without additional purification steps, such as bleaching. Like the emulsification properties, other researchers have exploited the fact that galactoglucomannan often carries associated lignin fragments, where the lignin component has been utilized as a crosslinker to enhance the properties of microfibrillated cellulose composite materials [156]. In addition to micro- and nanocellulose composites, glucomannan has been used as a component with graphene oxide to form interesting cryogels involving directional freezing and subsequent pyrolysis [157]. The cryogels had extremely high surface area and superior adsorption capacity for organic solvents and oils. In addition, food packaging has been investigated for mannan use as well, either in water-cast plasticized films [158] or blends with other biobased polymers, such as gelatin [159] or chitosan [160]. Like many other polysaccharides, galactoglucomannan, can be readily chemically modified, enhancing film properties [161], tuning modulus and yield at break. Other chemical modification methods have tried to create functional block co-polymers to use as surfactants for enhanced interactions with cellulose [162]. These applications show the diversity of mannans uses after designing interesting derivatives. However, one additional approach is to use highly purified mannan with limited side-chains; Chanzy and co-workers showed highly interesting shish-kebab structures of crystals from ivory nut mannan nucleated on cellulose microfibrils. This study provided a bottom-up approach to create unique materials that could be integrated with synthetic and nano-based technologies [163].

### Cellulose

Cellulose is a homopolymer of β-1,4-linked d-glucopyranosyl residues that accounts for 40–50% of the wood in gymnosperms and angiosperms [111, 164]. To date, seven polymorphs of cellulose have been described (Iα, Iβ, II, III_I_, III_II_, IV_I_, and IV_II_) based on their crystal structures, from which Iα and Iβ are the only synthesized in Nature [165].

Cellulose (Fig. 3) is the major component of plant cell walls and is essential for preserving their structural integrity [166]. In recent years, cellulose biosynthesis has received unprecedented interest as an alternative to replace the conventional petroleum-based materials due to its unique properties, including high strength and stiffness, relatively low density, simple structure, tunability, and abundance [166]. Currently, cellulose is considered a promising source of precursor for the production of biopolymers, biofilms, packaging materials, insulating materials, electrical sensors, and biomedical scaffolds [102].

#### Cellulose engineering

Cellulose crystallinity is considered one of the major technical hurdles to make lignocellulosic biomass conversion a cost‐effective process [167, 168]. Hydrogen bonds between the β‐1,4‐glucan chains, and dispersive bonds between the planar ring faces determine cellulose crystallinity and limited solubility [169]. The relative amount of crystalline material in cellulose is normally measured by the cellulose crystalline index (CrI) [170]. Highly crystalline cellulose has a tight structure among associated glucan chains, and limits the space for enzymes and chemicals to hydrolyze the cellulose during biomass conversion and provides incredible mechanical integrity along the fibre axis [167]. For example, different studies have demonstrated that completely amorphous cellulose is hydrolyzed faster than crystalline or partially crystalline cellulose [167, 168]. Hence, genetic modifications that could affect crystallinity are of significant interest for improving biomass processing.

Different genetic alterations have been shown to affect cellulose synthesis, and have manifested in reduced cellulose crystallinity. For example, in Arabidopsis, a point mutation in the C-terminus of Cellulose Synthase 3 (CesA3, the *irregular xylem1-2* [*ixr1‐2*] mutant) showed reduced cellulose crystallinity (by 34%) when compared with wild-type plants without significant effects on plant growth (Table 1). A marked improvement in saccharification was shown in these mutants, where fermentable sugar release was 151% higher than the corresponding controls [168]. Similar results have been observed when the C-terminus of the CesA1 protein was mutated in Arabidopsis [171, 172]. However, modifications of *CesA* genes in trees have not shown similar positive impacts on reducing cellulose crystallinity. In poplar, *PtrCesA4*, *PtrCesA7-A/B* and *PtrCesA8-A/B* genes were characterized as the cellulose synthases responsible for the synthesis of secondary cell wall cellulose [173], and RNAi-suppression of these genes exhibited significant decreases in cellulose content, reductions in growth, collapsed vessels, and thinner fibre cell walls, without changes in the CrI [173].

Another approach to reducing cellulose crystallinity involves targeting cortical microtubules, as microtubules play an important role in guiding the movement of the cellulose synthase complex (CSC) during cellulose synthesis. The abundance and stability of the microtubules have been inversely correlated with the degree of cellulose crystallinity [174]. In Arabidopsis, a Microtubule-Associated Protein (RIC1) promotes microtubule bundling, and overexpression of the corresponding gene reduced the degree of cellulose crystallinity in plants [174]. It has also been found that microtubule mass is important for the secretion of other polysaccharides in the vicinity of the CSCs [175]. The presence of these ultrastructural features restrains the cellulose microfibrils growth and increases the amorphous content. When the microtubule mass is reduced, cellulose crystallinity increases as a consequence of the reduced frequency of structural anomalies in the microfibrils [175].

Genetic manipulation of endonucleases that have been suggested to contribute to the synthesis of cellulose also affects cellulose microfibril deposition. Downregulation of orthologs of *Korrigan* (*KOR*) in poplar (*PtrCel9A6* and *PtrKOR1*), an endo-1,4-β-d-glucanase have met with varying results, but in most cases caused a collapsed xylem vessel phenotype and reduced cellulose content and ultrastructure while affecting hemicellulose levels [176, 177]. Downregulation of the *KOR* ortholog in gymnosperms manifested in drastically reduced growth, but no changes in cellulose content [178]. In contrast, the overexpression of *KOR*-like genes in Arabidopsis (*PttCel9A1/KOR1*) increased cellulose content, decreased cellulose crystallinity, and improved glucose yields [179]. Similarly, the overexpression of two endo-β-1,4-glucanases in rice, *OsGH9B1* and *OsGH9B3*, notably reduced the degree of cellulose polymerization and CrI, and increased bioethanol yields [180].

Finally, as a strategy to increase cellulose synthesis in biomass, changes in the allocation of carbon from photosynthesis to increase UDP-Glu availability for the biosynthesis of cellulose has also been explored [181–184]. For example, overexpression of a cotton *Sucrose Synthase* (*SuSy* or* SUS*) gene in poplar enhanced cellulose content by 2–6% and increased wood density and cellulose crystallinity without influencing plant growth [185]. Similarly, the overexpression of *OsSUS3* in rice enhanced cellulose content, but reduced CrI by 7–10% and xylose–arabinose proportions by 9–29% [186]. Taken together, these studies suggest that altering carbon skeleton availability in plants might also be a useful strategy to increase cellulose production/synthesis, and may at the same time affect cellulose ultrastructure and, therefore, has potential applications for cellulose and fibre industries.

While cellulose has been industrially used throughout history, recent scientific endeavours have focused on nanotechnology, generating significant advances in numerous fields. This research focus stems from early studies on na nocellulo se materials that showed significant reinforcement to lightweight polymer composites [187]. In these applications, aspect ratio (length/diameter) of the nanocellulose material was shown to be critical. Moreover, novel cellulose nanocrystals derived from tunicates revealed the upper limits of composite reinforcement, as tunicate-derived nanocrystals displayed outstanding aspect ratios and formed a desired percolating network [188]. Polymer latex materials were shown to exhibit increased stiffness of the rubbery plateau by two orders of magnitude [189], with later studies showing on-demand modulus change, acting as stimuli-responsive materials [190]. Plant-derived nanocelluloses have their reinforcement potential tied directly to aspect ratio; hence, cellulose engineering strategies should provide cellulose nanocrystals with exceptional dimensions, either through changing the crystallinity or the degree of polymerization of the native cellulose polymer. In addition, this can be best illustrated when examining nanocelluloses derived from different plant sources, and reported on cellulose nanocrystals (CNCs) [191] properties and oxidized nanofibrillated celluloses [192]. For the latter, correlation between the surface charge and crystallite size was established indicating cellulose microfibril dimensions *in planta* will influence the isolated nanocellulose dimensions. However, even with the different novel nanocellulose dimensions, primary production (pilot or demonstration scale) continues to focus on wood fibre as the primary feedstock. As such, controlling cellulose microfibril synthesis in trees, the current primary feedstock for nanocellulose production, should be closely evaluated to help optimize size and isolation efficiency for future materials applications.

Importantly, further development of nanocellulose use has seen promise for wide ranging applications including photonic films [193], catalyst templates [194], drug delivery [195], medical diagnostics [196], food emulsions [197], and in carbon fibre production [198]. Part of the functionality relates to the ability of nanocelluloses, especially CNCs, to form lyotropic liquid crystal suspensions [199]. As these films are dried, they maintain their highly organized chiral nematic structure. Anisotropic phase formation [200], pitch, and spacing are influenced by a number of parameters including size of the crystals (Fig. 4) [201]. Furthermore, transparency and haze for nanofibrillated cellulose are impacted by the size of the cellulose fragments [202]. Finally, diffusional rotation is impacted by size of the particle, which is a key parameter [203] in bottom up alignment of cellulose nanoparticles into strong fibres using flow focusing devices [204]. Controlling the molecular weight and crystallinity of cellulose will provide a starting material with a greater toolbox for cellulose nanoparticle size and geometry which impacts their potential applications.

### Lignin

In its native state, lignin is a co-polymer of mixed aromatics, with the majority of the basic units derived from three canonical monolignols: *p*-coumaryl, coniferyl, and sinapyl alcohol [205]. Different families of plants have different types and concentrations of these units, as illustrated in Fig. 2. Hardwoods and grasses produce all three monomers (G, S, and *p-*hydroxyphenyl [H] units) during lignification and vary in concentration depending on species and growing environment, while softwoods are largely S deficient and composed almost exclusively of G units (~ 95 of 100 aromatics). The S/G ratio of hardwoods on average ranges from 2 to 3, while grasses containing more H than most woody species. Significant reviews have provided details of these features, but it is noteworthy to highlight the extent to which the degree of substitution of the ortho positions on the aromatic rings will impact the resulting distribution of interunit linkages, as well as potential application of the lignin, vide infra. Although these canonical monolignols are dominant, they do not represent all the fundamental building blocks of lignin, as 11 different classes of lignin monolignols have now been discovered as more species, tissues, and cell types have been thoroughly examined with state-of-the-art spectroscopic technologies [206].

The lignification process, controlled via an “end-wise” polymerization process, was first modelled in vivo by Freudenberg in the 1950s [207]; however, despite the significant research efforts that followed, no single model of lignin currently exists. Common linkages within the polymer have, however, been identified, and include aryl ether (β-*O*-4), dibenzodioxin (5-*O*-4), resinol (β–β), spirodienone (β-1), and phenylcoumaran (β-5) (Figs. 2, 3). Whole cell wall analysis of native lignin has provided a better understanding of native lignin, and more importantly how lignin changes during various isolation procedures [208–210]. Employing a combination of ^13^C and 2D ^1^H–^13^C HSQC NMR analyses, 84 (100 Ar units) chemical linkages in lignin have been clearly labelled and analyzed quantitatively with a rank: β-*O*-4 (45–84) > β–β (1–11) > β-5 (3–12) > 5–5(1–7) > β-1(1–9) > 4-*O*-5 (minor) [211, 212]. Native-like lignin can be treated as a hydroxyl rich substance with 90–160 total hydroxyl groups per 100 aromatic C9 units. This number is relatively high, because even with the phenolic hydroxyl groups of the monolignols involved in the majority of the linkages found in lignin, the native *C*_γ_ hydroxyl is preserved during polymerization. The repeat unit becomes further oxygenated at the *C*_α_. This carbon is highly electrophilic immediately after the formation of the β-*O*-4 linkages during the polymerization process; at which time water is reacted at this benzylic carbon and forms a secondary hydroxyl group, while acids or alcohols from neighbouring hemicelluloses can bond and form block co-polymers with lignin [148]. These structures enhance the difficulty of extracting lignin from the cell wall matrix, reducing the efficiency for such processes as paper production or enzymatic saccharification in bioethanol production. Historically, forest industries have bred for trees that have low lignin contents to help processing and now the field of plant biotechnology is providing new ways to approach this problem. Moreover, it is becoming increasingly apparent that the inherent plasticity of the lignification process in plants can form the foundation for the synthesis of several very unique and valuable aromatic compounds, and more importantly the collection of potential compounds continues to increase and may only be restricted by biosynthetic pathway limitations.

#### Increased process efficiency, but reduced biomass yield

Over the past two decades, almost every known pathway gene (Fig. 2), independently or in combination, associated with secondary cell wall lignification has been augmented, using a variety of different genetic engineering tools [20, 41]. Although many of these approaches have been successful in reducing lignin content and/or composition, several resulted in various forms and extents of growth retardation, including reduced stem height, reduced diameter, and pendant phenotypes [213, 214]. Thus, although an increased processing efficiency per gram biomass has been witnessed, an overall product yield improvement may not be evident, as the total biomass recovered could be deleteriously affected. For example, downregulation of *4-coumarate:Co-A ligase* (*4CL*) [215], *p-hydroxycinnamoyl-CoA:quinate/shikimate p-hydroxycinnamoyltransferase* (*HCT*) [216], and *CCR* [217] in poplar resulted in growth impairments, such as a reduced diameter and reduced height compared to wild-type plants. In alfalfa, downregulation of *cinnamate 4-hydroxylase* (*C4H*), *p-coumarate 3-hydroxylase* (*C3′H*) and *HCT* also resulted in a yield penalty [218, 219]. In recent years, via a multi-omics integration study, it was shown that severe growth reduction manifesting from downregulation of some of the phenylpropanoid gene families does not necessarily correlate with lignin content [216]. For example, downregulation of *phenylalanine ammonia-lyase* (*PAL*) had a lignin content of only 9.4% (versus ~ 23%) while maintaining similar growth patterns as wild-type trees.

Several very thorough and informative studies showed that there are different factors that contribute to the observed growth reduction that commonly occur during downregulation of the core lignin biosynthetic pathway genes, including: (i) collapsed vessels; (ii) transcriptional reprogramming; and/or (iii) accumulation of inhibitors or toxic intermediates of the phenylpropanoid pathway. Moreover, it is possible to (almost completely) complement the yield penalty by expressing the aforementioned respective genes under a vessel-specific promoter in the mutant [220–222]. In that way lignification in the vessels is restored, while the lignin content of the fibres remains low, and the saccharification efficiency of the transgenic plants is, therefore, still increased relative to wild-type trees. With the recently published CRISPR-TSKO technology [223], it should be relatively easy to translate these observations to several, if not all biomass crops, as this technology can specifically target different cell types, tissues, and organs in the plant [223]. The second factor that contributes to the growth reduction is based on the involvement of two subunits (MED5a and MED5b) of the Mediator complex (*i.e.*, a transcriptional co-regulator that is crucial for both basal and regulated eukaryotic transcription) [224] in the phenylpropanoid homeostasis in Arabidopsis [225]. By stacking the *med5a*/*med5b* double mutant with the *ref8-1* mutant (*c3h* mutant), the growth and lignin amount are almost completely restored in the *med5a*/*med5b ref8-1* triple mutant compared to wild type and *med5a/med5b*. Most likely, the change in one or more wall-bound or soluble phenylpropanoid compounds disturbs the homeostasis of the phenylpropanoid pathway due to the block in C3′H in *ref8-1* mutant [226]. This change resulted in a transcriptional cascade that would lead to repression of lignification and reduced growth. By mutating *med5a/med5b* this transcriptional cascade is blocked in the *med5a/med5b ref8-1* triple mutants. Interestingly, the lignin generated in these plants is composed of nearly purely H units, which alone could have major implications in value-added polymer production, as polymeric lignin composed of only H units is naturally linear in structure [226]. The *Med5a/Med5b* subunits seem to be conserved among different plants species [225, 226], which is an essential condition to translate this strategy to biomass crops. The third hypothesis of dwarfism in certain lignin mutants is the hyperaccumulation or hypoaccumulation of intermediates or derivatives of the phenylpropanoid pathway that are essential or toxic for the plant. In association with the synthesis of monolignols, there are other biosynthetic pathways branching from the main phenylpropanoid pathway, such as flavonoid and suberin biosynthesis, and some enzymes for the biosynthesis of salicylic acid are shared with the phenylpropanoid pathway [219, 227]. For example, in Arabidopsis and alfalfa that have reduced HCT activity, the content of salicylic acid is negatively correlated with the stem height, which leads to the hypothesis that the hyperaccumulation of salicylic acid might be the reason for dwarfism [219, 227], but this mechanism may be unique for HCT [228].

**Fig. 5:**
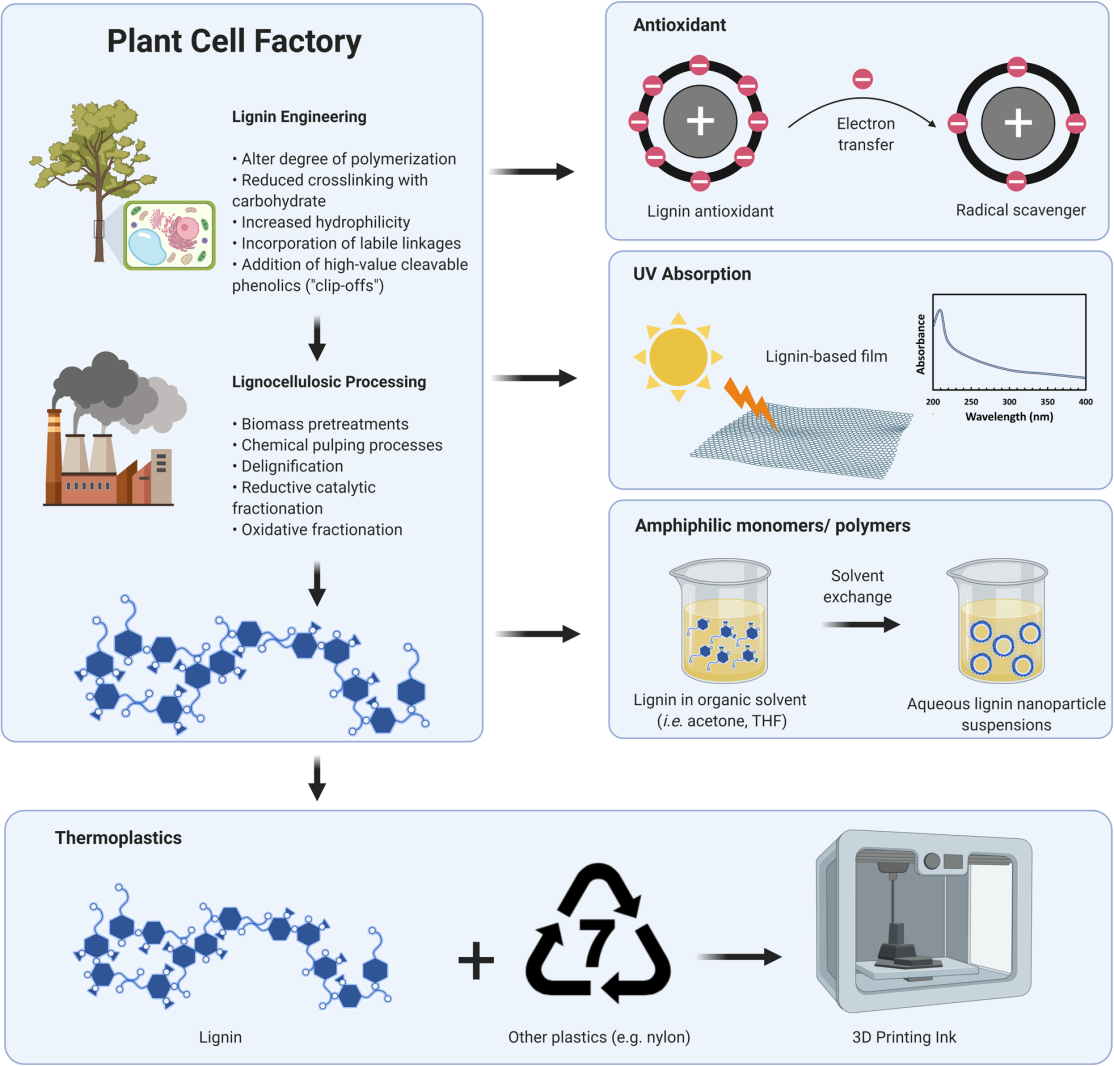
Schematic flow diagram highlighting that both plant engineering and down-stream lignin fractionation approaches can and need to be combined to isolate high purity lignin fractions with preferred structural characteristics for industrial utilization. Properties inherent in the lignin can be exploited for functional materials that include, but are not limited to, antioxidants, UV absorptive films, self-assembled nanoparticles, and melt processable lignin for 3D printing

To summarize, reduced activity of phenylpropanoid enzymes can result in yield penalties. These yield penalties are not desired for the overall biorefinery, as more land will be needed to produce the required biomass and thus the costs associated with biomass procurement will be higher. However, not all targets will manifest in altered growth phenotypes, and there are different strategies, as highlighted previously, to complement this yield penalty. Importantly, however, these strategies need testing in true biomass crops, and the plants need to be grown in the field, as these translational studies remain vital to bring such biotechnological approaches to fruition. Moreover, a reduction in lignin amount is likely not sufficient to optimize lignocellulosic biomass for the biorefinery, as it forms a potential vital carbon skeleton for several advanced application (highlighted below). Thus, augmenting lignin structurally may be a more useful target to improve the ease of extraction and consequently enhance recovery for advanced material applications (Figs. 4, 5).

#### Lignin engineering

Along with the aforementioned reductions in total cell wall lignin content, the lignin composition can be altered in such a way that it is easier to extract the lignin from the lignocellulosic biomass. To increase the value and the ease of use of lignin, there are different ways to strategically change the lignin composition, for example create lignin that exhibit (i) shorter lignin chains; (ii) less cross-linking with carbohydrates; (iii) increased hydrophilicity; and (iv) incorporation of labile linkages (Fig. 5) [205]. Depending on the desired end product(s) of a biorefinery (e.g., will the lignin be used as a polymer for the production of materials, or degraded to monomers for the production of specialty chemicals), different types of interunit lignin linkages may be desired. As such, lignin can be designed in such a way that there is an increase in the monomer yield, facilitate the delignification process, and/or provide novel, value-added monomers. Below we provide some examples of these strategies (Table 1 for summary), and comment on potential uses/applications for the designed lignin fractions (Fig. 5).

i. Reduced degree of polymerization
  Reducing the degree of polymerization would instinctively result in easier separation of lignin from the carbohydrates. As such, shortening the lignin polymer can be achieved by altering the type, distribution, and timing of the in planta synthesis of monomers that are only capable of initiating or terminating the polymerization process. Examples of monomers that solely act as chain-starting monomers include hydroxybenzenoids and its derivates [229], hydroxyarylpropanols (e.g., dihydroconiferyl alcohol in gymnosperms) [230], and the flavanol tricin [231]. To this end, it has been shown that the degree of polymerization can be reduced in Arabidopsis lignin by expressing the bacterial gene, *Hydroxycinnamoyl-CoA Hydratase-Lyase* (*HCHL*), in lignifying cells. No biomass yield penalty or a reduced lignin content was apparent in the transgenic plants, and lines with intermediate HCHL activity showed increased saccharification efficiency [229]. The opposite effect has been observed in the natural maize mutant, *C2-Idf*, that displays only a residual expression (5%) of the *Chalcone Synthase* (*CHS*) gene, one of the key enzymes in the biosynthesis of flavonoids and thus tricin. In these mutant maize plants, there is a reduced incorporation of tricin in the lignin polymer and the lignin was enriched in β–β and β-5 units [232], which is an indication that tricin is indeed only an initiator of lignin polymerization, and due to the absence of tricin, more monolignol dimerization reactions occur as opposed to lignin chain initiation [231, 233]. These examples illustrate that reducing the degree of polymerization of the lignin polymer could be a good strategy to increase process efficiencies, but also serve as a means to produce and isolate lignin-derived oligo-polymers that could be used in the synthesis of materials and polymeric materials.
  A potential application for an enriched pool of oligo-polymers would include a source of antioxidants, an important class of compounds that can stabilize free radicals (Fig. 5). For example, Pouteau and co-workers blended 15 different types of technical lignin with polypropylene which is typically used in food packaging applications [234], and showed that the low molecular weight lignins had stronger antioxidant activity in polypropylene. This result was similar to other polyphenolic compounds, *i.e.*, tannin or flavonoids, as the antioxidant activity of lignin arises from the phenolic hydroxyl groups and ortho methoxy groups. Moreover, the lower molecular weight (low degree of polymerization) lignin had a larger content of free phenolics, which was correlated to antioxidant activity [234, 235]. Tagami et al. also investigated antioxidant activity of both softwood and hardwood fractionated lignin, and found lower molecular weight samples outperformed the high molecular weight samples, with the hardwood showing a better response than two known radical scavengers, rutin and quercetin [236]. Antioxidant activity has also been demonstrated in other polymer classes, such as polyesters. Kai et al. prepared a lignin–polyester copolymer, followed by electrospinning, to prepare a biocompatible and antioxidant tissue engineering material [237]. The antioxidant capacity increased with the addition of lignin concentration and even shown biocompatibility with in vitro tests. With a growing market in natural polyphenolics, lignin is, therefore, an ideal candidate to replace the synthetic antioxidants currently used in plastics, food, and healthcare applications.
ii. Less cross-linking with carbohydrates
  Along with the non-covalent bonds adjoining the different polymers of the plant cell wall, it has been proposed there are also covalent bonds between lignin and hemicellulose [123]. For years this was only observed in grasses, where ferulic acid substitutions of the arabinoxylan backbone could be oxidized (via unspecified laccases and peroxidases or via oxidized monolignols), creating radicals of feruloylated xylan, which could then couple with radicals of a growing lignin polymer, resulting in a cross-linked matrix of polysaccharides and lignin [123, 238]. With this biochemical knowledge, efforts to reduce the amount of ferulic acid synthesized in the cell wall have been attempted. For example, the maize mutant *seedling ferulate ester* (*sfe*) has reduced feruloylation, resulting in better forage digestibility [239–241]. Another example is the rice knock-out mutant *xax1*, a *family 61 glycosyl transferase* (*gt61*), which is responsible for the arabinosyl substitutions of the xylan backbone. These mutant lines display increased processing efficiency, as might be expected, but the mutants were dwarfed [124].
  The cross-linking between hemicellulose and lignin via arabinoxylans that are substituted with ferulic acid is unique to grasses, as eudicots do not have arabinosyl substitutions on the xylan, and gymnosperms arabinosyl substitutions are also most likely not feruloylated. However, it has been shown that there are ether bonds between galactoglucomannan and lignin in Japanese red pine [148]. For eudicots it has been proposed that hemicellulosic alcohol and carboxylic groups can act as an alternative nucleophilic donor for the re-aromatization of the quinone methide intermediates that occur during lignification at the β position, and this could lead to an ether or ester linkage between the hemicellulose and lignin. By incorporating *o*-diphenol groups, such as caffeyl alcohol (incorporated in the seedcoat in various members of the Cacacea family) [242] and 5-hydroxyconiferylalcohol, which can undergo internal trapping of the quinone methide intermediate resulting in benzodioxane structures, there will be no change partner for external nucleophilic donors. In support of these concepts, mutations or downregulation of *COMT* results in the incorporation of 5-hydroxyconiferyl alcohol in the lignin polymer and thus a concurrent rise in benozdioxane structures in the lignin [243]. Although there is no associated reduction in lignin, *comt* mutants display increased saccharification efficiency, which could be a consequence of the reduced covalent bonds between hemicellulose and lignin due to the incorporation of 5-hydroxyconiferylalcohol [244].
  Tailored plants with increased ease of separation would be desirable as it improves processing efficiency especially in obtaining valuable building blocks, such as nanocelluloses mentioned above. The traditional biomass isolation route, aimed at obtaining high-purity carbohydrate substrates, always induces unfavorable condensed linkages in lignin and subsequent coupling to the cell wall when treating lignocellulosic biomass with chemicals at high temperatures. As such, the resultant lignin has an unknown and unpredictable structure, which limit the materials that can be derived to relatively low value. The “lignin-first” strategy is aimed at retaining as much of the native lignin structure as possible, that Nature perfected over millenia, without sacrificing the value of other cell wall chemical components. One interesting approach combined both enzymatic saccharification and bead milling for the production of fermentable sugars with native-like lignin residuals, simultaneously. This process, named simultaneous enzymatic saccharification and comminution lignin (SESC lignin), was capable of recovering abundant lignin with the potential for a variety of different applications [245, 246]. Shikinaka et al. adopted this SESC lignin (originated from straw and cedar) and blended it with clay materials to produce non-flammable and moisture-permeable UV protective films. Their results revealed that species differences give rise to different structures that impacted the UV absorbance properties. Straw SESC lignin contained tricin and *p*-coumarate esterified units, which led to films displaying a wide spread in π conjugation bonds with improved UV absorption properties (Fig. 5) [245]. Processes, such as SESC, would be more effective without lignin carbohydrate linkages and would enhance the final solubility of the residues, which usually contain some component of polysaccharides.
iii. Increased hydrophilicity
  The phenolic polymer lignin is primarily hydrophobic. Incorporation of monomers that are more hydrophilic compared to the canonical monolignols would result in lignin polymers that are: (i) more soluble in water; (ii) engage in fewer hydrophobic interactions in the cell wall; and (iii) display improved enzyme accessibility to the carbohydrates during saccharification [205]. Monomers with increased hydrophilicity contain additional hydroxyl groups or are substituted with hydrophilic moieties, such as carbohydrates or polyol groups [247]. Based on artificially lignified maize cell walls, incorporation of highly hydrophilic monomers, such as sinapoyl glucose, coniferyl and sinapyl glucosides, and rosmarinic acid indeed resulted in reduced cell wall lignification [247]. Such an engineering strategy in biomass crops would not only likely improve cell wall processing, but also may effect plant development. Therefore, increased hydrophilicity of monomers can be an alternate strategy; however, the alternative monolignol should not be too hydrophilic.
  Enhanced hydrophilicity of lignin should lead to improved amphiphilic properties of isolated lignin. Recently, new lignin applications based on its ability to self-assemble has garnered increasing interest in the scientific community. Li et al. [248] adopted kraft lignin as a stabilizer to form an oil/water fuel emulsion, based on both its high calorific value and its abundance. To improve the solubility of lignin in neutral water, the lignin was first carboxymethylated which unfortunately requires toxic reagents. Being able to design lignin with enhanced hydrophilicity would provide a route to promote these kinds of applications without the need for chemical modification. In addition, an enhanced amphiphilic nature of isolated lignin would further lend itself for assembly into interesting colloidal lignin nano- to micro-scale particles (Fig. 5). These colloidal spheres can be prepared by either solvent exchange [249, 250] or aerosol techniques [251]. The diameter of the hollow spheres is controllable by the dilution rate in the solvent/water system [252]. Furthermore, taking advantage of the native properties of lignin,* i.e.*, UV absorbing, antioxidant, and antibacterial properties, the value of these uniform colloidal particles could provide the basis for novel coatings [253], controlled release applications [254], and even drug delivery systems [255]. An example how these biobased materials can serve functions for environmental remediation, Bharti and collaborators [256] used lignin particles with heavy alcohol to demonstrate excellent absorption properties in the recovery of oil spill. Hence, more hydrophilic lignins within improved amphiphilic properties could help the self-assembly properties of these materials for advanced applications.
iv. Incorporation of labile linkages
  If a high monomer yield of phenylpropanoids is desired in the biorefinery [e.g., via reductive catalytic fractionations (RCF)], the lignin polymer should have a low fraction of uncleavable carbon–carbon bonds [257]. As sinapyl alcohol lacks a free *ortho*-position, it cannot form the 5–5 and β-5 interunit bonds during radical coupling, which means that lignin that is rich in S units (*i.e.*, hardwoods) contain fewer uncleavable C–C bonds. By increasing the S/G ratio in grasses and softwoods, the monomer yield obtained by RCF could be improved. For example, by overexpressing *F5H* in poplar, the amount of S units in the lignin polymer was shown to be more than 90% [40]. This resulted in a monomer yield of 77% after hydrogenolysis of the transgenic lines, while the monomer yield of the native wild-type poplar was 24.5% [258]. Along with the increased monomer yield, incorporation of labile bonds in the polymer backbone may also lead to improved ease of lignin extraction under milder condition, and thus more efficient separation of the lignin from the carbohydrates [35, 259] in both pulping [35] and biofuel pretreatments [259]. Similarly, *CCR*-deficient plants have easily cleavable acetal bonds due to the incorporation of ferulic acid in the lignin polymer [217, 260]. Besides perturbations in the phenylpropanoid pathway, alternative monolignols could be introduced in the cell wall via heterologous gene expression. For example, a *Feruloyl-CoA:Monolignol Transferase* (*FMT*) from *Angelica sinesis* was expressed in poplar trees, creating the so called “*zip*-lignin” that have ester linkages in the lignin backbone, which is inherently ether-linked, due to the incorporation of ferulate conjugates. These trees have improved cell wall digestibility after mild alkaline pretreatment and an increased pulping efficiency [261, 262]. Likewise, the expression of a *Brachypodium distachyon p-Coumaroyl-CoA:Monolignol Transferase* (*PMT*) gene in Arabidopsis and poplar resulted in the incorporation of high-value pendant *p*-coumarate conjugates into the lignin, which resulted in a higher frequency of terminal units with free phenolic groups [263, 264]. Due to these free phenolic groups, the engineered Arabidopsis lignin is more soluble in alkali at room temperature [264]. Similarly, curcumin (diferuloylmethane) was recently introduced in Arabidopsis’ lignin via simultaneous expression of the *Curcuma longa’s* genes *Diketide-CoA Synthase (DCS)* and *Curcumin Synthase 2* (*CURS2*). Curcumin, when incorporated in the lignin polymer, can be hydrolytically degraded under alkaline conditions at low temperatures due to its β-diketone functionality and thus result in biomass with an improved saccharification efficiency [265]. Finally, Arabidopsis that are modified with the bacterial Cα-dehydrogenase that oxidizes the α-hydroxyl groups in lignin, resulted in the appearance of chemical labile α-keto-β-ether units [266].
  The engineering of labile linkages within lignin creates polymers that may be lower in molecular weight. Lignins such as lower molecular organosolv hardwood lignin or some solvent fractionated lignins have reduced glass transition temperatures (*T*_g_) and often can be transformed into a liquid-like state during heating [267]. Other structures also helped to lower the *T*_g_ of the hardwood lignin, such as the presence of additional methoxy groups; together these aliphatic ethers provide higher degrees of freedom including rotation and bending modes of the functional groups and segments. Ultimately, these structures help the lignin to be more suitable for ink extrusion for 3D printing applications. To make additive manufacturing processes greener, many renewable polymers have been investigated as potential filaments for this process. As lignin is one of the only components to flow at certain temperatures without further derivatization, this cell wall polymer would be the best candidate for bio-based fuse-deposition filaments for 3D printing (Fig. 5). For evaluating printability, the shear rate and temperature-dependent rheological parameters and the thermal conductivity of a polymer play key roles in a polymer filament. Along these lines, Nguyen et al*.* developed 3D printable materials with 40–60% lignin with nylon and carbon fibres [268]. This was a function of the dominance of the β-*O*-4′ linkage retained in organosolv hardwood lignins, along with its original aliphatic propyl side chain, making the lignin flexible. This result was in contrast to lignin derived from softwoods, which were more condensed due to the dominance of the G units. Another example of using lignin with high S-content was organsolv lignin isolated from hybrid poplar. After acylation, these lignins were highly soluble in commercial stereolithography resins to form block co-polymers [269]. The authors targeted lignin as a new photopolymer resin for 3D printing material as its renewable and contained several UV absorptive moieties (e.g., aromatic rings, and C=O bonds). As a result, the optimum amount of modified lignin-based resins showed improved ductility with lower UV dosage, but decreased thermal stabilities compared with commercial resin. The key to forming these lignin materials was low molecular weight with readily modifiable hydroxyl groups.

#### Partial delignification

Alternatively, a recent scientific advance has demonstrated unique materials created from partially delignified wood with intact cellular structure [270]. Research in functional wood materials covers several areas of enhanced mechanical strength; functional properties, such as magnetism, thermal control, and UV shielding. These have important function in building structures, and functionalized wood has also been used in membranes for environmental remediation, energy storage, and flow bioreactors. Based on this approach of selective delignification, optically transparent composites were created from low lignin content woods with low density, including balsa and basswood [271, 272]. The delignified wood maintained its original microporous structures and were subsequently filled with refractive index matching polymer to become optically transparent. The highly orthotropic structure of the ensuing wood composite had interesting light transmission properties with different light scattering angles. Especially, the combination of high transmittance and haze characteristics, providing a route to obtain both natural sunlight with good thermal insulation and some privacy (haze). Furthermore, such delignified wood can be used as a multifunctional material, including electronic and in photonic devices [273]. However, drawbacks exist in the preparation of transparent wood, including preparation time and the inherent toxicity of delignification. Li et al., showed an effective delignification process with alkaline treatment with maintaining high transmittance and haze properties of materials [274]. This process selectively removed only chromophores of lignin, such as coniferaldehyde, but preserved the mechanical stability of delignified wood compared with conventional methods that employ sodium chlorite. Hence, lignin that can be readily leached from lignocellulosic biomass without impacting the cellulose structure would create a significant opportunity to scale these materials to larger dimensions.

#### The ideal lignin monomer: from plant’s needs to society’s demands

Lignin has enabled plants to grow over 100 m in height by strengthing the polysaccharide-rich scaffold running throughout the cell wall [275]. The very nature of the monolignol precursor to lignin has been critical in providing the structural performance required by the plant cell wall to enable these feats. The canonical phenylpropanoid units that form the monomers for most lignin, must be considered a performance engineered ‘ideal’ structural unit with the right balance of stiffness, flexibility, and hydrophilicity for this role as a thermosetting matrix material in this advanced hygroscopic nanocomposite structure known as the cell wall (technically xylem). This does not preclude the fact that lignification can be augmented to obtain higher value products.

To date, more than 160 alternative monomers have been identify that could lead to altered processing efficiency, with at least one of the aforementioned functionalities [276]. We purport that the ideal lignin monomer for biomass processing would have a combination of functionalities. For example, putative monomers that have a hydrophilic compound, a chemically labile bond, and two *o*-diphenol groups (resulting in less links between hemicellulose and lignin) may provide unique lignin and lignin–carbohydrate interactions. Such “monomers” have been tested via artificially lignified maize cell walls, such as epigallocatechin and rosmarinic acid [247, 277, 278]. Both of these monomers show potential as alternative monolignol, as they were able to form radicals, polymerize into lignin, and show improvements in saccharification (although, as mentioned, the lignification is reduced when rosmarinic acid is used as an alternative monomer) [277, 278]. However, there are challenges that must be overcome for success in engineering (some) of these potential monolignols in planta, keeping in mind that the compound needs to be produced in sufficient concentrations inside the cell (regulatory and detoxification constraints), that the alternative monomer needs to be efficiently transported to the cell wall, where they should be able to undergo radical and combinatorial coupling efficiently, and that all without having a major influence on plant development.

Furthermore, along with improved processing efficiency, the ideal lignin monomer should provide added-value to the lignin polymer in such a way it could be used for novel lignin-derived high-value materials production, or in a way that it can compete with existing petroleum-derived products. For example, the observed increased concentrations of aldehydes in *CAD*-deficient plants [230, 244, 279, 280], which have an increased saccharification efficiency [244, 280], could be used for different applications, as coniferylaldehyde for example possesses pesticidal and antibacterial activity to kill and control insects [281]. Another example of such a potential monolignol is caffeyl alcohol, which is present in the seedcoat of *Vanilla planifolia* [242] and various Cactaceae [282], creating C-lignin. Polymerization of caffeyl alcohol in C-lignin results in a linear lignin polymer, that is composed of benzodioxane units and, therefore, naturally possesses reduced cross-linking between the different polymers of the cell wall. C-lignin has a unique acid-resistant feature, which also means that it maintains its native structure after pretreatment, and if subject to hydrogenolysis will result in simple monomeric catechols units [283]. These catechols units could then be used for the synthesis of high-value chemicals and polymeric materials.

## Conclusion—combining biotechnology and material science

How can we create a bio-based economy, where we grow, convert, and use renewable carbon in an economically, socially, and environmentally sustainable manner is a question of our generation. Fortunately, we are in the dawn of the age of synthetic biology after years of investment in a variety of life science “omics” projects. From this body of research, there are a number of enabling technologies and high-throughput tools that provide for rapid identification of active genes that show promise to drive better utilization of biological resources. As highlighted above, the plant cell wall is a key source of polymeric materials and nanostructures that are useful for potential sustainable consumption. While existing components of the cell wall can be isolated as simple sugars through hydrolysis, we can and should be able to exploit useful modifications of the plant cell wall polymers for extraction and application. Furthermore, synthetic polymer science has been developed based on the toolbox of organic synthesis available to chemists. Plant, as well as microbrial biomass, provide a new pathway to design new monomers that may be too difficult to produce in a reactor or are unavailable with traditional reactions. From this standpoint polymers with new performance may be able to be created from custom-designed feedstocks. By genetically engineering the starting lignocellulosic biomass, or other breeding technologies enabled through biotechnology, potentially other high-value compounds could be incorporated *in planta*, and after depolymerization and/or isolation (e.g., saponification with mild base) could be used directly as high-value chemicals and new monomers. For example, in *CAD*-deficient plants aldehydes [230, 279, 280] accumulate, while caffeyl alcohol is naturally incorporated in the seedcoat of various Cactaceae [282] and *Vanilla planifolia* [235]. In contrast, plants can be strategically designed to produce high-value, easily accessible compounds, *i.e.*, *p-*coumarate, by spatially and temporally expressing genes, such as *PMT*, that facilitates the incorporation of pendant *p-*coumarate into plant lignins [263, 264], acting as a value-added “clip-off” phenolic. This phenolic compound has been used to directly make polystyrene replacements by making a hydroxyethyl derivative and subsequently polymerizing into a polyethylene coumarate [284]. Hence, high-value biological compounds can be used directly as precursors in a variety of polymeric applications [285, 286]. As both a reduced total lignin content and lignin with defined alternative monolignols could lead to an increased processing efficiency, stacking such traits could facilitate game-changing outcomes. For example, in Arabidopsis it has been shown that combining a low-lignin mutant with a *comt* mutant (reduced cross-linking), results in synergistic effects on saccharification yields and provide routes for facile lignin extraction of wood templates for functional materials [287]. Another example is overexpressing the galactan synthase gene (*GALS1*), the *UDP-*galactose/UDP-rhamnose transporter (*URGT1*) and the UDP-glucose epimerase (*UGE2*), leading to a fourfold increase of pectic galactan. In addition, this increased galactan trait was engineered into vessel-complemented xylan-deficient Arabidopsis plants (high galactan and low xylan plants) and finally, these traits were stacked with a low lignin trait obtained by expression of the bacterial *Quinate and Shikimate Utilization B* (*QsuB*) gene. This strategically engineered plant resulted in an increase in saccharification efficiency and a 33-fold increase of the hexose/pentose ratio in Arabidopsis without causing a yield penalty [288]. Such creative designs highlight one of many possibilities, and provides the driving motivation for gene stacking as an attractive strategy to design bioenergy crops that are tailor-made for biorefinery applications. The combinations and targets are endless, and as such, so are the potential applications and polymer products that could be produced. As we move forward with the ability to isolate plant-based biopolymers, fermentable sugars, and new monomers with preferred structures for the creation of useful materials, nearly all carbon within the plant will be captured in the process as useful materials, minimizing waste.

## Data Availability

All data generated or analyzed during this study are included in this published article.

## Abbreviations

[Me]GlcA: (Methyl)glucuronic acid
4CL: 4-Coumarate:CoA ligase
AaXEG: *Aspergillus aculeatus* xyloglucanase
AtBGAL10: β-Galactosidase 10
ARAF: Arabinofuranosidases
Araf: Arabinofuranose
AX: Arabinoxylans
C3H: *p*-Coumarate 3-hydroxylase
C4H: Cinnamate 4-hydroxylase
CAD: Cinnamyl alcohol dehydrogenase
CCoAOMT: Caffeoyl-CoA *O*-methyltransferase
CCR: Cinnamoyl-CoA reductase
CesA: Cellulose syntase
CHS: Chalcone synthase
CNC: Cellulose nanocrystals
COMT: Caffeic acid *O*-methyltransferase
CrI: Cellulose crystalline index
CSC: Cellulose synthase complex
CSE: Caffeoyl shikimate esterase
CSLA: Glucomannan 4-beta-mannosyltransferase
CURS2: Curcumin synthase 2
DARX: Deacetylase on arabinosyl sidechain of xylan
DCS: Diketide-CoA synthase
ESK1: Eskimo1
F5H: Ferulate 5-hydroxylase
FMT: Feruloyl-CoA:monolignol transferase
GalA: Galacturonic acid
GALS: Galactan synthase gene
GAUT: Galacturonosyltransferase
GAX: Glucuronoarabinoxylans
G unit: Guaiacyl unit
G-layer: Gelatinous-layer
GlcA: Glucuronic acid
GT61: Family 61 glycosyl transferase
GUX: Glucuronyltransferase
GX: Glucuronoxylans
H unit: *p-*Hydroxyphenyl unit
HCHL: Hydroxycinnamoyl-CoA hydratase-lyase
HCT: *p*-Hydroxycinnamoyl-CoA:quinate/shikimate *p*-hydroxycinnamoyltransferase
HG: Homogalacturonan
irx: Irregular xylem
KOR: Korrigan
MW: Molecular weight
NMR: Nuclear magnetic resonance
PAL: Phenylalanine ammonia-lyase
PCWs: Primary cell walls
PL: Pectate lyase
PME: Pectin methylesterase
PMEI: Pectin methylesterase inhibitor
PMT: *p*-Coumaroyl-CoA:monolignol transferase
QsuB: Quinate and shikimate utilization B
RCF: Reductive catalytic fractionations
RG-I: Rhamnogalacturonan I
RG-II: Rhamnogalacturonan II
Rha: Rhamnose
RIC: Microtubule-associated protein
S unit: Syringyl unit
SCWs: Secondary cell walls
SESC lignin: Simultaneous enzymatic saccharification and comminution lignin
SFE: Seedling ferulate ester
SUS: Sucrose synthase
SuSy: Sucrose synthase
*T*_g_: Glass transition temperature
UGE: UDP-glucose epimerase
URGT: UDP-galactose/UDP-rhamnose transporter
XEH: XyG:endohydrolase
XET: XyG:endotransglycosylase
XTHs: Xyloglucon endotransglycosylase/hydrolase
XyG: Xyloglucan
Xyl: Xylose
